# Primary SARS-CoV-2 variant of concern infections elicit broad antibody Fc-mediated effector functions and memory B cell responses

**DOI:** 10.1371/journal.ppat.1012453

**Published:** 2024-08-15

**Authors:** Karlijn van der Straten, Denise Guerra, Gius Kerster, Mathieu Claireaux, Marloes Grobben, Angela I. Schriek, Anders Boyd, Jacqueline van Rijswijk, Khadija Tejjani, Dirk Eggink, Tim Beaumont, Steven W. de Taeye, Godelieve J. de Bree, Rogier W. Sanders, Marit J. van Gils

**Affiliations:** 1 Amsterdam UMC, location Academic Medical Center, Department of Medical Microbiology and Infection Prevention, Laboratory of Experimental Virology, Amsterdam, The Netherlands; 2 Amsterdam Institute for Immunology and Infectious Diseases, Amsterdam, The Netherlands; 3 Department of Infectious Diseases, Public Health Service of Amsterdam, Amsterdam, the Netherlands; 4 Stichting HIV monitoring, Amsterdam, the Netherlands; 5 Center for Infectious Disease Control, National Institute for Public Health and the Environment (RIVM), Bilthoven, The Netherlands; 6 Amsterdam UMC, location Academic Medical Center, Department of Internal Medicine, Amsterdam, The Netherlands; 7 Department of Microbiology and Immunology, Weill Medical College of Cornell University, New York, New York, United States of America; University of Virginia, UNITED STATES OF AMERICA

## Abstract

Neutralization of Severe Acute Respiratory Syndrome Coronavirus 2 (SARS-CoV-2) by human sera is a strong correlate of protection against symptomatic and severe Coronavirus Disease 2019 (COVID-19). The emergence of antigenically distinct SARS-CoV-2 variants of concern (VOCs) and the relatively rapid waning of serum antibody titers, however, raises questions about the sustainability of serum protection. In addition to serum neutralization, other antibody functionalities and the memory B cell (MBC) response are suggested to help maintaining this protection. In this study, we investigate the breadth of spike (S) protein-specific serum antibodies that mediate effector functions by interacting with Fc-gamma receptor IIa (FcγRIIa) and FcγRIIIa, and of the receptor binding domain (RBD)-specific MBCs, following a primary SARS-CoV-2 infection with the D614G, Alpha, Beta, Gamma, Delta, Omicron BA.1 or BA.2 variant. Irrespectively of the variant causing the infection, the breadth of S protein-specific serum antibodies that interact with FcγRIIa and FcγRIIIa and the RBD-specific MBC responses exceeded the breadth of serum neutralization, although the Alpha-induced B cell response seemed more strain-specific. Between VOC groups, both quantitative and qualitative differences in the immune responses were observed, suggesting differences in immunogenicity. Overall, this study contributes to the understanding of protective humoral and B cell responses in the light of emerging antigenically distinct VOCs, and highlights the need to study the immune system beyond serum neutralization to gain a better understanding of the protection against emerging variants.

## Author summary

The SARS-CoV-2 virus, first identified in 2019, swiftly led to a global pandemic. Crucial for the protection against re-infections are sufficient levels of neutralizing antibodies in the blood. However, SARS-CoV-2 have evolved into antigenically distinct variants of concern (VOC) that escape protection by these neutralizing antibodies. In addition, the relatively rapid waning of serum antibody titers raises questions about the sustainability of protection. In this study, we investigated the ability of antibodies and memory B cells to recognize the emerging variants after primary SARS-CoV-2 infection with different variants of concern. Compared with neutralizing antibodies, antibodies that can mediate Fc-mediated effector functions as well as memory B cells were, in general, more cross-reactive to other variants, irrespectively of the variant causing the infection. However, we found variations in the level of immune responses depending on the infecting variant, indicating differences in the immunogenicity of SARS-CoV-2 VOC. These results highlight the need to study the immune system beyond serum neutralization to gain a better understanding of the protection against emerging variants.

## Introduction

The continued evolution of Severe Acute Respiratory Syndrome Coronavirus 2 (SARS-CoV-2) raises concerns about the protection of infection- and vaccine-induced immunity against Coronavirus Disease 2019 (COVID-19). Evolving variants of SARS-CoV-2 were previously designated by the WHO as variants of concern (VOCs) in case of increased transmissibility, detrimental changes in COVID-19 epidemiology, increased virulence, and/or decreased effectiveness of public health measures, vaccines and/or therapeutics compared to preceding variants [1]. Based on this definition, the Alpha, Beta, Gamma, Delta, and Omicron sub-variants have been designated for some time as VOCs. Some of these variants have frequently caused re-infections and vaccine breakthrough infections due to insufficient levels of pre-existing protective immunity [2,3].

Neutralizing antibody (NAb) titers are a strong correlate of protection against symptomatic and severe COVID-19 [4–6]. The main target of NAbs is the spike (S) glycoprotein, which consists of a variable distal S1 subunit, that includes the N-terminal domain (NTD), two C-terminal domains (CTD1 and CTD2) and the receptor binding domain (RBD), and a more conserved membrane-proximal S2 subunit that contains the fusion machinery and the transmembrane domain [7–9]. Although NAbs targeting the NTD and the S2 subunit have been described [10,11], the most potent NAbs target the RBD and block the interaction with the human angiotensin-converting enzyme 2 receptor (ACE2) by binding to the receptor binding motif, by destabilization of the S protein, or by steric hindrance [12]. Re-infections by SARS-CoV-2 VOCs are likely to be caused by both the relatively rapid waning of NAb titers over time [5], and by differences in the antigenicity as a consequence of the high variability in the S protein amino acid composition between VOCs [13,14].

The escape of heterologous VOCs from NAb titers have been extensively described [15–17]. Especially the highly mutated Omicron BA.1 and BA.2 variants are substantially resistant to neutralization by serum antibodies elicited by other variants, resulting in the designation of Omicron as a different antigenic cluster compared to preceding VOCs [18–20]. In addition, the more recently emerged Omicron sub-variants, including Omicron XBB.1.5, XBB.1.16 and EG.5, further escape NAb responses and are resistant to clinically used monoclonal antibodies [21–23]. Despite the escape of VOCs from NAbs, a reduction in disease severity during re-infections is often observed [24,25]. This suggests additional, possibly more preserved protective immune mechanisms besides NAb titers, which may include antibody Fc-mediated effector functions and the presence of memory B cells (MBCs) that may respond upon antigen re-encounter.

Both neutralizing and non-neutralizing antibodies can mediate effector functions by binding free viral particles, or binding the antigen presented on the membrane of infected cells. Antigen-bound immunoglobulin (Ig) activates innate cells such as macrophages, neutrophils and natural killer (NK) cells expressing, among others, activating Fc-gamma receptor IIa (FcγRIIa) and IIIa (FcγRIIIa). Activation of FcγRIIa and FcγRIIIa results in cell lysis and clearance of infection via phagocytosis (ADCP) and antibody dependent cellular cytotoxicity (ADCC), respectively [26]. The protective and therapeutic potential of antibody Fc-mediated effector functions has been previously suggested for multiple viral pathogens, including human immunodeficiency virus 1 (HIV-1), dengue virus, influenza virus, and respiratory syncytial virus (RSV) [27–30]. In the context of SARS-CoV-2, Fc-mediated effector functions demonstrated significant importance to prevent severe COVID-19 [31–34], and contribute to the effectiveness of COVID-19 vaccines [35–37]. However, high levels of S protein-specific serum IgG with enhanced Fc-mediated effector functions were detected in sera of critically ill COVID-19 patients, illustrating the fine balance between the desired pro-inflammatory status of the immune system and over-activation resulting in a cytokine storm and severe COVID-19 [38]. The antibody Fc-mediated effector functions elicited by COVID-19 vaccines have been shown to be more preserved compared to serum neutralization [39]. In addition, Fc-mediated effector functions mediated by convalescent sera have been studied for D614G, Beta and Delta-infected patients, where differences were found in the breadth of this antibody response between the VOCs causing the infection [40].

Furthermore, as the serum antibody titers decline overtime, it is also of primary importance to study the capacity of the immune system to respond to antigen re-encounter by reactivation of previously induced MBCs. Upon an initial exposure, naive B cells proliferate and differentiate into antibody secreting cells or MBCs [41–44]. These MBCs can undergo new rounds of affinity maturation within germinal centers upon antigen re-encounter. In the light of an increasing number of re-infections with antigenically distinct variants, elicited MBCs should ideally cross-react to slightly diverse antigens, and differentiate during the germinal center reaction into highly potent and specific antibody-secreting cells. This desired recall response has been shown in mRNA vaccinated patients, where an Omicron BA.1 breakthrough infection caused proliferation and differentiation of wild-type (WT)-induced MBCs towards cross-reactive RBD-specific MBCs able to bind both the WT and Omicron BA.1 RBD [45]. To the best of our knowledge, it remains unknown whether RBD-targeting MBCs induced by primary infection with a VOC are also able to cross-react with heterologous RBDs and whether the breadth of the MBC response differs between the VOC causing the infection.

In this study, we examined the magnitude and breadth of S protein-specific antibody Fc-mediated effector functions of convalescent sera, and the RBD-specific MBC responses following a primary D614G, Alpha, Beta, Gamma, Delta, Omicron BA.1 or Omicron BA.2 infection. We observed a preserved interaction of anti-S protein serum antibodies with FcγRs, which very strongly correlated with the S protein-specific IgG responses. In contrast, the RBD-specific cross-reactive MBC response did not always mirror the serum responses. This was especially the case for the Delta and Alpha-elicited B cell responses. The preservation of components of the adaptive immune system other than serum neutralization, and the distinct immune responses elicited by VOCs, highlight the need to study the immune system beyond neutralization in order to gain a better understanding of the immune protection against emerging SARS-CoV-2 variants.

## Results

### Study population

This study used a cohort of 67 primary, PCR-confirmed SARS-CoV-2 VOC infected patients with no history of any COVID-19 vaccination. We previously described the patient characteristics and serum neutralizing antibody responses [18]. Following the previously defined inclusion criteria of either a sequence confirmed, or a high likelihood of SARS-CoV-2 VOC infection, this cohort included 20 D614G-, 12 Alpha-, 8 Beta-, 4 Gamma-, 11 Delta-, 8 Omicron BA.1- and 4 Omicron BA.2-infected patients. The minority of patients (n = 19, 28%) required hospitalization for their COVID-19, of which nine were infected with D614G, nine with Alpha, and one with Delta. The median age of the cohort was 42 years (IQR 29–54 years). Serum and peripheral blood mononuclear cells (PBMCs) were collected 3 to 11 weeks after symptom onset (median 40 days; IQR 35–45 days).

### S protein-specific IgG titers strongly correlate with the level of FcγR-interaction

**Fig 1:**
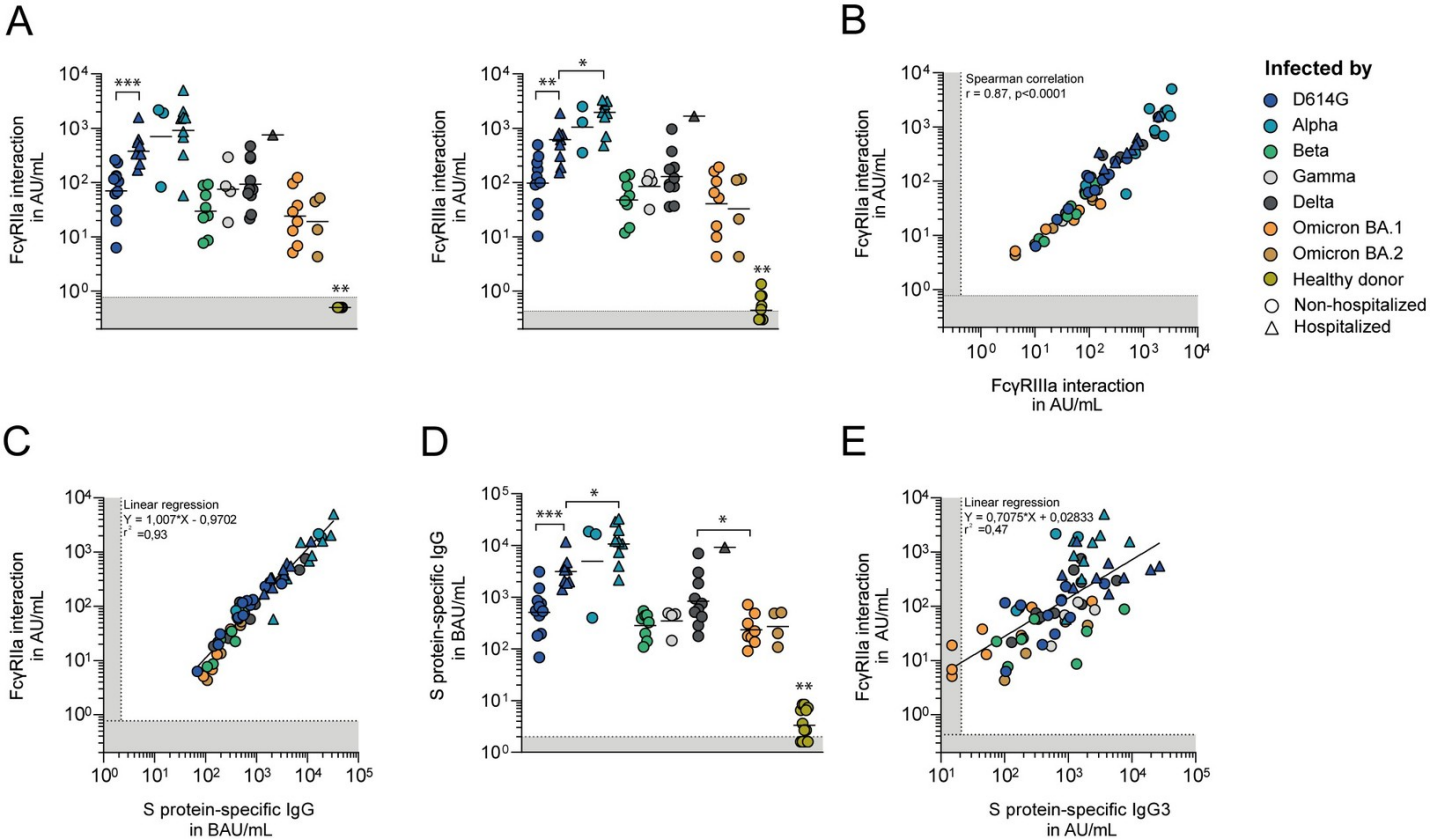
Antibody responses following primary SARS-CoV-2 VOC infections, including level of interaction with FcγRIIa and FcγRIIIa. A) Level of interaction of S protein-specific serum antibodies with FcγRIIa (left panel) and FcγRIIIa ectodomain dimers (right panel) expressed as arbitrary units per mL (AU/mL). Convalescent sera were grouped by VOC causing the infection, and separated based on COVID-19 disease severity. Bars indicate the geometric mean level of interaction. A Mann-Whitney U test with Benjamini-Hochberg correction was used to compare titers between the following VOC groups: all non-hospitalized groups with more than three patients, hospitalized and non-hospitalized D614G-infected patients, hospitalized D614G- and Alpha-infected patients, and to all convalescent sera groups with pre-pandemic sera. Only statistically significant differences are reported (* = q<0.05, ** = q<0.01, *** = q<0.001). The gray bar indicates the lower limit of quantification. B) Spearman correlation between the level of interaction with FcγRIIa and FcγRIIIa by autologous S protein-specific serum antibodies. C) Linear regression analysis between the level of interaction with FcγRIIa, and the S protein-specific IgG titers in binding antibody units per mL (BAU/mL). D) S protein-specific IgG titers in BAU/mL, analyzed in the same manner as Fig 1A. E) Linear regression analysis between the level of interaction with FcγRIIa and the S protein-specific IgG3 titers in arbitrary units per mL (AU/mL).

We assessed the Fc-mediated effector functions ADCP and ADCC, by measuring the interaction of autologous S protein-specific serum antibodies with FcγRIIa and FcγRIIIa ectodomain dimers, respectively [46], using a standardized in-house Luminex multiplex assay [47,48]. The highest geometric mean level of interaction with FcγRIIa and FcγRIIIa were found for the D614G-, Alpha- and Delta-infected groups (Fig 1A). All hospitalized patients were infected with one of these three variants. Among D614G-infected patients, the level of interaction with FcγRIIa and FcγRIIIa was 6- and 5-fold higher in sera of hospitalized compared to non-hospitalized patients, respectively (Mann-Whitney U test with Benjamini-Hochberg correction, q = 0.0003 and q = 0.0028, respectively), suggesting an association between COVID-19 disease severity and the level of FcγRIIa and FcγRIIIa interaction. Therefore, we separated the non-hospitalized from the hospitalized patients (Fig 1A). An increased level of FcγR-interaction for hospitalized patients was also observed for the Alpha- and Delta-convalescent sera, but here group sizes preclude any statistical comparisons. Interestingly, the S protein-specific serum responses of both the hospitalized and non-hospitalized Alpha-infected patients showed a remarkably strong interaction with FcγRIIa and FcγRIIIa. These differences remained when accounting for sparse data using Bayesian statistics (S1A Fig). Due to the association between COVID-19 disease severity and the level of interaction with FcγRIIa and FcγRIIIa, we then restricted comparisons between VOC groups to convalescent patients with comparable COVID-19 disease severity. Among hospitalized patients, the convalescent sera of the Alpha-infected group exhibited a 3.4-fold stronger interaction with FcγRIIIa, and to a lesser extent with FcγRIIa (2.2-fold), compared to the D614G-infected group (q = 0.028 and q = 0.12, respectively) (Fig 1A). Among non-hospitalized patients, convalescent sera of Beta, Omicron BA.1 and Omicron BA.2 patients showed a 2.4 to 4.9-fold and 2.0 to 3.9-fold lower interaction with FcγRIIa and FcγRIIIa, respectively, compared to D614G-, Gamma- and Delta-infected patients, albeit not statistically significant. The levels of interaction with FcγRs were significantly higher for all VOC groups compared to pre-pandemic healthy donor sera.

The level of interaction with FcγRs by convalescent sera was highly comparable between FcγRIIa and FcγRIIIa, resulting in a strong and significant correlation (Spearman correlation, r = 0.87, p<0.001) (Fig 1B). Few sera showed a relative preference to interact with FcγRIIa or FcγRIIIa. These patients were all infected by different strains, indicating a preserved ratio in the level of interaction with FcγRIIa and FcγRIIIa by autologous S protein-specific serum antibodies among VOCs.

IgG is the most abundant antibody isotype present in serum. Therefore, we studied whether the variation in FcγRIIa and FcγRIIIa interaction between convalescent groups mainly reflects the differences in S protein-specific IgG titers, or whether some VOCs are more prone to elicit FcγR-interacting antibodies. We found that the S protein-specific IgG titers nearly perfectly correlated with the level of interaction with FcγRIIa (linear regression, r^2^ = 0.93) and FcγRIIIa (r^2^ = 0.92) (Figs 1C and S1B, respectively). The correlation did not appear to numerically differ between the VOC patient groups, implying that the level of interaction with FcγRIIa and FcγRIIIa by autologous S protein-specific serum antibodies is mainly dependent on serum IgG titers for all VOCs.

### SARS-CoV-2 VOCs elicit quantitatively different serum antibody responses

It remains unclear whether differences in S protein-specific serum IgG titers between VOC groups are inherent to the VOC causing the infection, or are mainly dependent of other study- or patient-specific differences between the groups, such as timing of sampling and the ability to mount an antiviral antibody response. The days post symptom (DPSO) (i.e. timing of sampling) did not significantly correlate with the S protein-specific IgG titers (Spearman correlation, r = -0.12, p = 0.32) (S1C Fig). Additionally, the serum IgG response against the fusion protein of RSV (RSV-F) (Kruskal-Wallis test, p = 0.80) or hemagglutinin of influenza virus (HA) (p = 0.13) did not differ between the VOC groups, suggesting that the ability to mount an antibody response to viral proteins in general does not differ (S1D Fig). Furthermore, these RSV-F- and HA-targeting serum IgG responses did not significantly correlate with age (Spearman correlation, r = -0.01, p = 0.93 for RSV-F, and r = 0.04, p = 0.73 for HA, respectively) (S1E Fig). Together, we found lacking evidence that any study- or patient-specific characteristics substantially contributed to differences in S protein-specific IgG titers between the convalescent VOC groups.

To test whether these differences in S protein-specific serum IgG responses between VOC groups reflect generally stronger SARS-CoV-2-specific antibody responses, including antibodies against other structural proteins, we measured serum IgG binding to the more conserved nucleocapsid (N) protein of the WT strain (S1F Fig). We then compared these responses with the S-protein specific IgG titers (Fig 1D). A comparable 6.0- and 5.3-fold difference was observed for the S protein- and N protein-specific IgG responses between hospitalized compared to non-hospitalized D614G-infected patients (q = 0.0007 and q = 0.02, respectively). Additionally, in non-hospitalized patients, the Beta and Omicron BA.1 convalescent sera showed the lowest response, while D614G and Delta convalescent sera showed stronger S protein- and N protein-specific IgG responses (Figs 1D and S1F). Notably different between the S protein- and the N protein-specific IgG responses, is the N protein-specific serum binding profile of non-hospitalized Alpha-infected patients, which no longer exhibits one of the strongest responses. These findings held when accounting for sparse data using Bayesian statistics (S1G Fig).

Overall, the differences between convalescent VOC groups in the interaction with FcγRs by S protein-specific serum antibodies are strongly associated with COVID-19 disease severity, while there was lacking evidence that other patient- or study-specific characteristics substantially contributed to this difference. Nevertheless, differences in the antibody response between VOC convalescent groups still remain present when considering the COVID-19 disease severity.

### SARS-CoV-2 VOCs elicit a qualitatively different serum antibody response

To further explore the relationship between S protein-specific IgG titers and the level of interaction with FcγRs, we examined the S protein-specific IgG subtype responses. First, we measured the S protein-specific IgG3 response, as this IgG subclass is known for its high affinity for FcγR on effector cells, and is thus of particular interest when studying Fc-mediated effector functions [49,50]. The S protein-specific serum IgG3 titers correlated less strongly with the level of interaction with FcγRIIa (Fig 1E, linear regression, r^2^ = 0.47) and FcγRIIIa (S1H Fig, r^2^ = 0.43) compared to the correlations found for the serum IgG titers (Figs 1C and S1B, respectively). This suggests that another subclass of IgG rather than IgG3, is primarily responsible for the interaction with FcγRIIa and FcγRIIIa. Next, we measured the S protein-specific IgG1 responses in a subset of samples, as IgG1 is the most abundant IgG subclass in serum. We observed a similar correlation between S protein-specific IgG1 levels and FcγR-interaction (S1I Fig, linear regression, r^2^ = 0.44 and r^2^ = 0.35 for FcγRIIa and FcγRIIa, respectively), indicating that both IgG1 and IgG3 subclasses interact to a similar degree with FcγRs in our Luminex assay. The correlation between the S protein-specific IgG subclasses and FcγR-interaction differed between VOC groups. The correlation between IgG3 titers and FcγR-interaction were numerically higher for Alpha convalescent sera compared to other VOC groups, while no such difference between VOC groups was observed for the correlation between IgG1 and FcγR-interactions. Indeed, when calculating the IgG3/IgG ratio, the Alpha group showed a statistically significant lower IgG3/IgG ratio compared to all other VOC groups, except compared to the Omicron BA.1 convalescent sera (S1J Fig). In contrast, the Gamma convalescent sera exhibited a statistically significant higher IgG3/IgG ratio compared to the Alpha, Delta, and Omicron BA.1 VOC groups. The ratios did not statistically significantly differ between the sera of hospitalized and non-hospitalized D614G-infected patients (p = 0.55), indicating that COVID-19 disease severity is unlikely to have substantially influenced this IgG3/IgG ratio in this cohort. Thus, qualitative differences in the antibody response between convalescent VOC groups were found, which cannot be directly linked to the variation observed in the level of FcγR-interaction.

### Antibody Fc-mediated effector functions are more cross-reactive than serum neutralization

**Fig 2:**
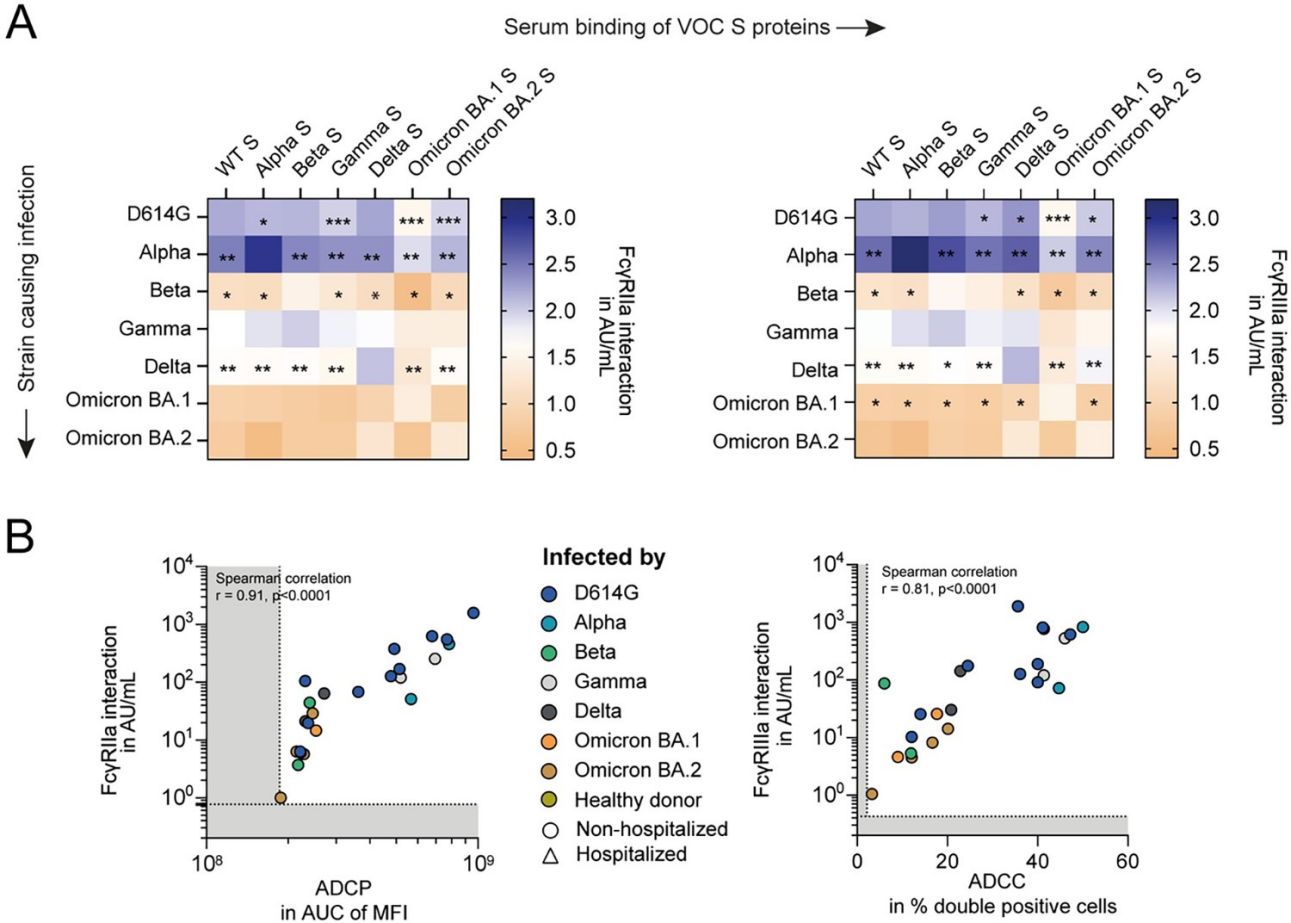
Cross-reactive S protein-specific IgG strongly correlates with the antibody Fc-mediated effector functions. A) Heatmap depicting the level of interaction with FcγRIIa (left panel) and FcγRIIIa (right panel) of autologous and heterologous S protein-targeting serum antibodies in logarithmic transformed Arbitrary Units per mL (AU/mL). A Mann-Whitney U test with Benjamini-Hochberg correction is used to compare the heterologous with the homologous S protein responses. Only statistically significant differences are reported (*q<0.05, **q<0.01, ***q<0.001). B) Spearman correlation comparing the interaction with FcγRIIa (left panel) or FcγRIIIa (right panel) by wild-type S protein-specific serum antibodies with functional antibody dependent cellular phagocytosis (ADCP) and cytotoxicity (ADCC) assays, respectively. ADCP is expressed as the area under median fluorescent intensity (MFI) curve (AUC); ADCC is expressed as the percentage of double positive (CD107^+^, IFNγ^+^) natural killer cells.

Next, we studied the cross-reactivity of the S protein-specific serum antibodies that interact with FcγRIIa and FcγRIIIa. Fig 2A shows a heatmap of the interaction with FcγRIIa (left panel) and FcγRIIIa (right panel) by autologous and heterologous S protein-binding serum antibodies (S2A and S2B Fig, respectively). Within each VOC group, the pattern of variation in the heterologous S protein-specific interactions with FcγRIIa and FcγRIIIa were highly comparable. Indeed, the ratio between these FcγR-interacting responses ranged up to a maximum of 2-fold, which did not differ substantially from the ratios found for the autologous S protein responses between FcγRIIa and FcγRIIIa (S2C Fig).

The breadth of the FcγR-interacting S protein-specific antibody responses was further studied by comparing the heterologous response to the autologous response in the same individual within a VOC group. Overall, the level of S protein-specific antibodies with FcγR interaction was strongest against the autologous S protein (Figs 2A, and S2A and S2B). Except for Omicron BA.2 convalescent sera, all sera showed the largest reduction in binding against the Omicron BA.1 S protein for both FcγRIIa and FcγRIIIa-interacting antibodies. The fold-change against Omicron BA.1 S protein for FcγRIIa and FcγRIIIa-interacting antibodies was the strongest for Alpha (10.6 and 10.4-fold) and Beta-infected patients (8.6 and 8.0-fold), followed by the Delta (6.1 and 5.9-fold), D614G (4.5 and 4.4-fold) and Gamma-infected patients (2.4 and 4.1-fold). This reduction in antibody titers against Omicron is in line with the many additional S protein mutations in this VOC compared to the earlier variants, and thereby being antigenically distinct from the previously emerged variants [18,20]. We previously found a 10-fold, 52-fold, 6-fold and 6-fold drop in serum neutralization against Omicron BA.1 for the D614G-, Alpha-, Gamma- and Delta-infected patients, respectively, with only 22 out of 66 studied patients having any detectable serum neutralization against Omicron BA.1 [18]. Thus, compared to previously obtained cross-neutralizing antibody titers of the same cohort, the FcγR-interaction by S protein-specific antibodies are more preserved.

In line with the autologous S protein-specific serum responses, the cross-reactivity of the FcγR-interacting S protein-specific serum antibodies correlated strongly and significantly with the corresponding heterologous S protein-specific IgG titers (S3 Fig). In addition, we found comparable slopes across VOCs when regressing the heterologous on autologous responses (S2D Fig). Together, this suggest that level of FcγR-interaction by both autologous and heterologous S protein-specific antibodies are mainly dependent on serum IgG titers, without substantial differences in this correlation between VOCs.

We validated the use of FcγR-dimers as a proxy for antibody Fc-mediated effector functions, by comparing the level of interaction with FcγRIIa and FcγRIIIa with cellular ADCP and ADCC assays, respectively. A subset of convalescent VOC sera was tested for the presence of WT S protein-specific serum antibodies mediating ADCP and ADCC. We found a strong correlation between the level of FcγR-interaction and ADCP (Spearman correlation, r = 0.91, p<0.0001) and ADCC (r = 0.81, p<0.0001) by WT S protein-specific serum antibodies (Fig 2B).

Altogether, we found that Fc-mediated effector functions by S protein-specific antibodies, measured as the interaction with FcγRIIa and FcγRIIa, are more preserved among VOCs compared to serum neutralization and are highly dependent on IgG titers.

## VOCs elicit divergent frequencies of RBD-specific memory B cells

Because of the waning of antibody titers over time, we next studied whether VOCs differed in the frequency of elicited autologous RBD-specific MBCs, and characterized the cross-reactivity of these MBCs to other heterologous RBDs. For this analysis, we selected a subset of the cohort: 8 D614G-, 4 Alpha-, 4 Beta-, 8 Delta-, 4 Omicron BA.1- and 4 Omicron BA.2-infected patients based on the presence of a cross-reactive neutralizing antibody response and availability of material [18]. The autologous RBD-specific B cells were gated from the total CD19^+^ B cell population and further phenotyped into four subsets based on their surface expression profiles of IgD and CD27 markers: naive (IgD^+^CD27^-^), non-switched memory (IgD^+^CD27^+^), classical memory (IgD^-^CD27^+^) and double negative memory (IgD^-^CD27^-^) B cells (S4A Fig).

Given the observed association between the antibody response and COVID-19 disease severity, we studied whether the frequency of RBD-specific MBCs differed between hospitalized and non-hospitalized patients. In our cohort, hospitalized patients developed a significantly stronger B cell response (median 0.046% and 0.029% in the total CD19^+^ B cell population and classical MBC compartment, respectively) compared to non-hospitalized patients (0.018% and 0.006%, respectively) (p = 0.014 and p = 0.004, respectively) (S4B Fig). These frequencies are in line with approximately 0.05% of MBCs being RBD protein-specific after infection with the WT strain, or following mRNA vaccination [44,51–53].

**Fig 3:**
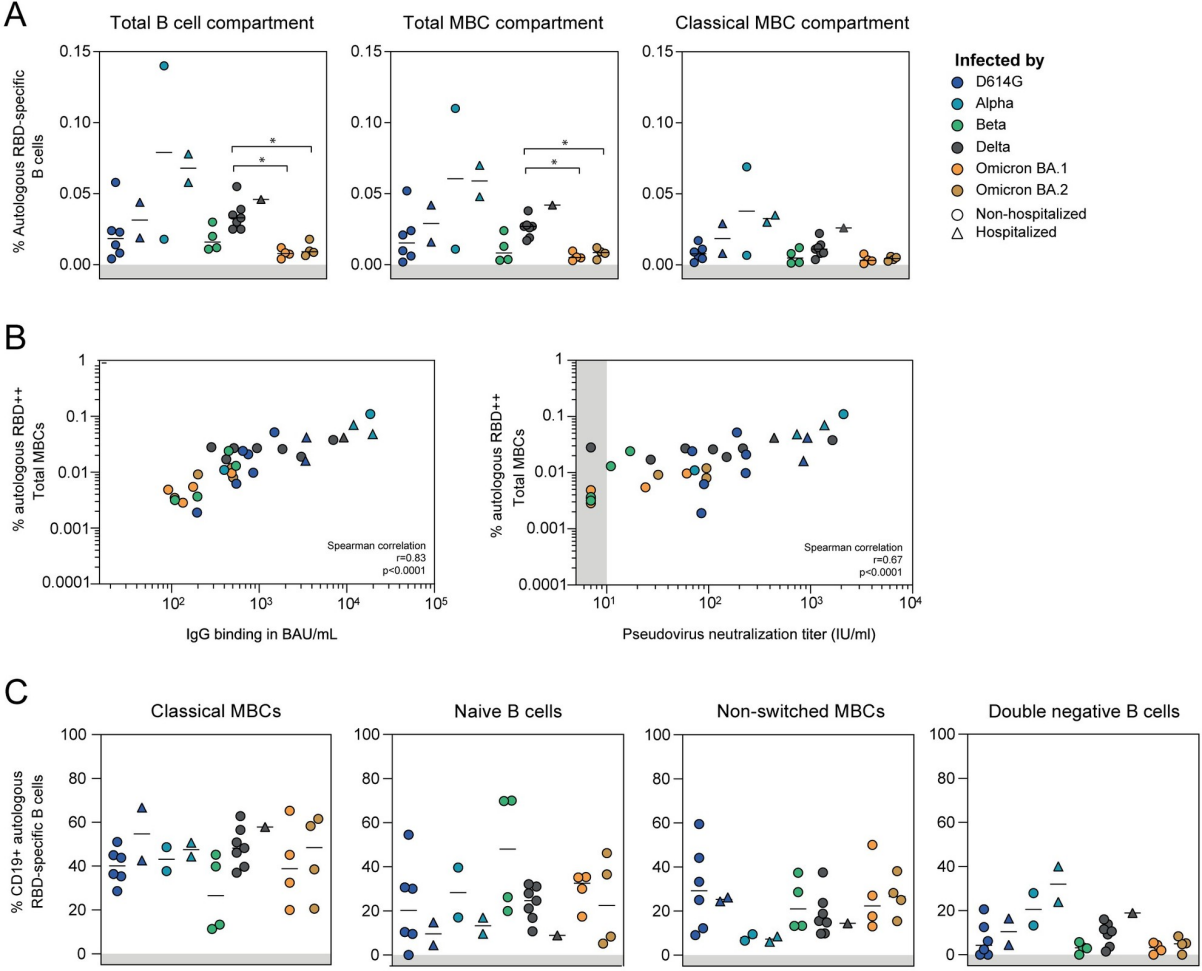
SARS-CoV-2 VOCs elicit a quantitatively and qualitatively different RBD-specific B cell response. A) Percentage of autologous RBD-specific B cells as part of the total CD19^+^ B cell (left panel), total memory B cell (MBC) (middle panel) and classical MBC (right panel) compartments. B cell responses were grouped by VOC causing the infection, and separated based on COVID-19 disease severity. Only the B cell responses from non-hospitalized patients were compared between VOC groups using a Mann-Whitney U test with Benjamini-Hochberg correction. Only significant differences are reported (* = q<0.05). Bars show median values for all groups. B) Spearman correlation between percentage of autologous RBD-specific total MBCs and S protein-specific IgG binding (left panel), and serum pseudovirus neutralization titers (right panel). C) From left to right: the phenotype of the RBD-specific classical MBC, naïve B cell non-switched MBC and double negative B cell compartments.

To limit the bias caused by the influence of disease severity on the frequency of RBD-specific MBCs, we next studied the differences between VOC groups in only non-hospitalized patients. For the non-hospitalized patients, the median frequencies of RBD-specific B cells in the total CD19^+^ B cell population ranged from 0.008% to 0.08% (Fig 3A, left panel). The two non-hospitalized Alpha-infected patients showed, despite the limited number of samples and the high variation observed, a trend towards a higher frequency (0.08%), followed by Delta (0.033%), D614G (0.018%), Beta (0.016%), Omicron BA.2 (0.009%) and BA.1 (0.008%) groups. Frequencies of autologous RBD-specific B cells in the Delta group were significantly higher compared to Omicron BA.1 and BA.2 groups (q = 0.046 in both comparisons). In addition, we assessed the frequency of autologous RBD-specific B cells within the total MBC compartment (including non-switched, classical, and double negative MBCs) (Fig 3A, middle panel), as well as in the classical MBC subset only (Fig 3A, right panel), and found the differences between VOC groups to be comparable with the total B cell compartment analysis. These findings held when accounting for sparse data using Bayesian statistics (S4C Fig).

We observed a significantly positive correlation between the percentage of these autologous RBD-specific total MBCs with S protein-specific IgG titers binding responses (r = 0.83, p<0.0001) and to a lesser extent with serum neutralization (Spearman correlation, r = 0.67, p<0.0001) (Fig 3B). When considering the subset of only classical MBCs, we found the correlation with serum neutralization to be slightly numerically higher (Spearman correlation, r = 0.72, p<0.0001, S4D Fig). Interestingly, in six patients (18.8%), we could detect RBD-specific total MBCs in the absence of detectable autologous serum neutralization (<10 IU/mL). These patients were infected by different VOCs (two with Beta, one with Delta, two with Omicron BA.1 and one with Omicron BA.2).

Taken together, we observed differences in the frequency of RBD-specific MBCs between the VOC causing the infection, which is consistent with differences in serum neutralization and S protein-specific IgG titers.

### Distinct phenotypes of RBD-specific memory B cells elicited by different VOCs

Because of the differences observed in the frequency of RBD-specific B cells between the VOCs, we next studied whether additional differences in the B cell phenotype exist between the convalescent VOC groups. First, we studied whether the phenotype of the RBD-specific B cells differed between hospitalized and non-hospitalized patients. We found hospitalized patients to have significantly higher levels of RBD-specific double negative MBCs (median 18.90%) and less naive B cells (9.57%) compared to non-hospitalized patients (5.13% for double negative MBCs and 27.9% for naive B cells) (p = 0.011 and p = 0.013, respectively) (S4E Fig). Again, to mitigate the bias caused by COVID-19 disease severity, we focused on non-hospitalized patients for further analysis on B cell phenotypes. Among the RBD-specific B cell compartments studied, classical MBCs were the predominant subset developed across all VOC groups, except for Beta-infected patients, accounting for 48%, 48%, 43%, 40% and 39% in the case of Omicron BA.2-, Delta-, Alpha-, D614G- and Omicron BA.1-infected non-hospitalized patients, respectively (Kruskal-Wallis p = 0.42 for non-hospitalized patients only) (Fig 3C). Despite the Omicron BA.1 and BA.2 variants showing the lowest frequencies of autologous RBD-specific B cells (Fig 3A), these variants did elicit a switched classical MBC response comparable to other VOC groups (Fig 3C). Despite not statistically significantly different from the other VOC groups, the predominant RBD-specific B cell subset in Beta-infected patients consisted of naive B cells (median 48.0%), in contrast to D614G- (20%), Alpha- (28%), Delta- (25%), Omicron BA.1- (33%) and BA.2-infected non-hospitalized patients (22%) (Kruskal-Wallis p = 0.68 for non-hospitalized patients only) (Fig 3C). This suggests that infection with the Beta variant is likely to result in a less mature B cell response. No statistically significant differences between VOC groups were found for the non-switched and double negative MBC subsets (Kruskal-Wallis test for non-hospitalized patients only, p = 0.24 and p = 0.12, respectively), although both hospitalized and non-hospitalized Alpha-infected patients have numerically lower levels of non-switched and higher levels of double negative MBCs compared to other VOC groups (Fig 3C). These findings held when accounting for sparse data (S5A Fig).

The differences observed in the phenotype of RBD-specific B cells was further reflected by the immunoglobulin isotype. Total RBD-specific B cells of non-hospitalized Delta-infected patients contained a B cell population of predominantly IgG^+^ B cells (median 54%), followed by non-hospitalized D614G (31%), Alpha (51%), Beta (28%), Omicron BA.1 (37%) and BA.2 (27%) groups (S5B Fig). Beta-specific B cells were mainly IgM^+^ (65%), reflecting once again the less switched response elicited by this variant. In addition, Omicron BA.2-infected patients had a remarkably high IgG^-^IgM^-^ population (33%), which is suggestive of an IgA isotype, although this frequency was not statistically higher compared to the other groups (Kruskal-Wallis test for non-hospitalized patients, p = 0.08). Similar trends were observed when considering frequencies of only switched MBCs (S5C Fig). Overall, the percentage of RBD-specific IgG^+^ correlated positively with the autologous serum neutralization (Spearman correlation, r = 0.59, p = 0.0004), while a negative correlation was observed with IgM^+^ B cells (r = -0.51, p = 0.0026) (S5D Fig). The number of DPSO at which samples were taken, significantly, but weakly correlated with the percentage of IgG^+^ RBD-specific B cells (Spearman correlation, r = 0.36, p = 0.04) (S5E Fig). We found no correlation with either the percentage of IgM^+^ RBD-specific B cells (r = -0.16, p = 0.38) or the percentage of autologous RBD-specific total MBCs (r = 0.04, p = 0.8) (S5E Fig).

Overall, in addition to quantitative differences between VOC-induced RBD-specific B cell responses, we found differences in the phenotype of these B cells, which could not be explained by differences in timing of sampling.

### D614G and Delta variants induce the most cross-reactive RBD-specific memory B cells

**Fig 4:**
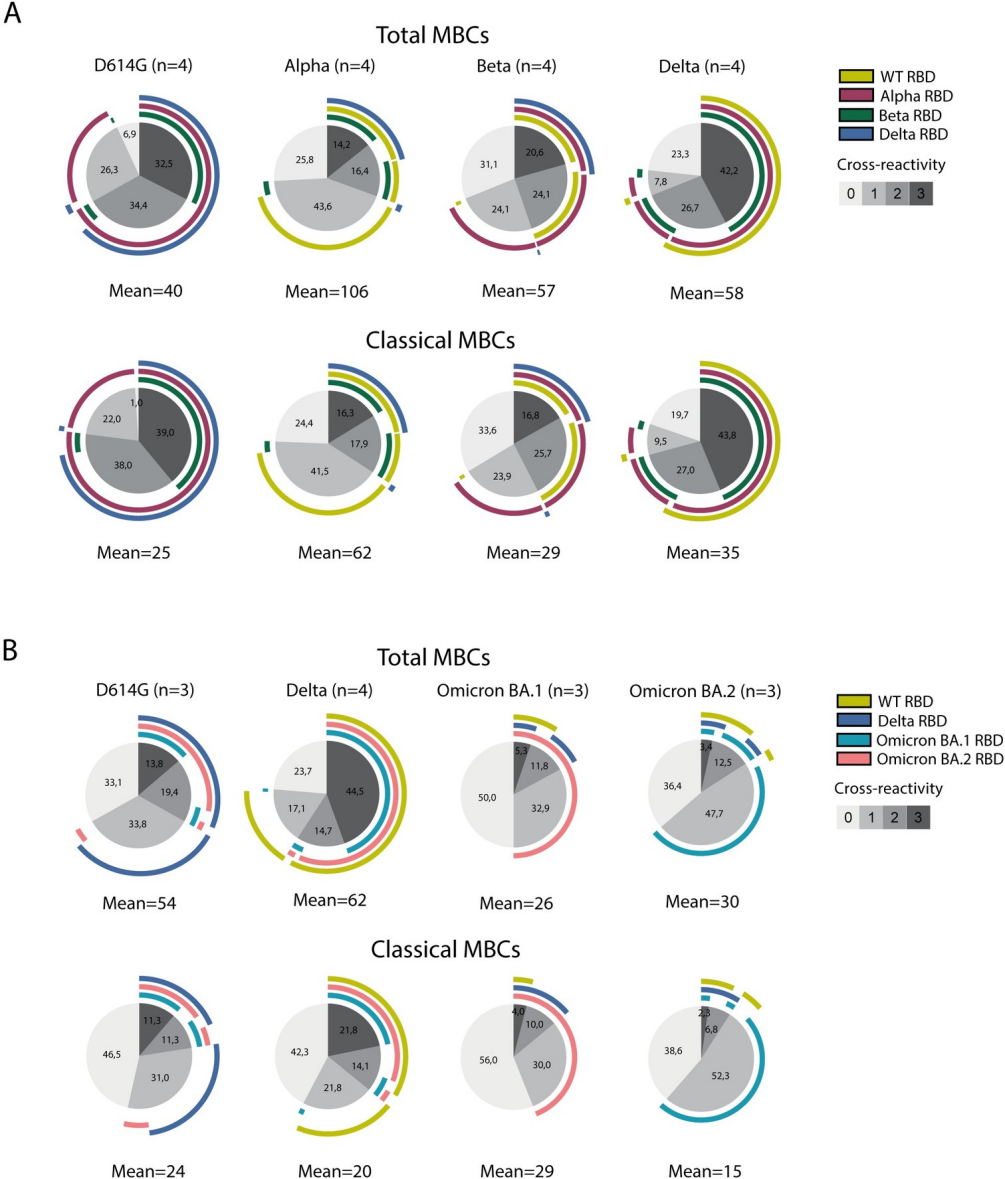
Breadth of MBCs elicited after primary SARS-CoV-2 VOC infections. A) Reactivity of total memory B cells (MBCs) (top panel) and classical MBCs (bottom panel) against the early-pandemic VOCs. The number of patients and the mean number of B cells used in this analysis are indicated on top and below the corresponding plots, respectively. The cross-reactivity legend indicates whether B cells bind only to the autologous RBD (0), or recognize one, two or three other heterologous RBDs. The proportion of B cells that are cross-reactive are written in the slices. The arcs represent the corresponding binding variant. B) Reactivity of total MBCs (top panel) and classical MBCs (bottom panel) against the later-pandemic VOCs, analyzed in the same manner as in A. See S1 Table for percentage heterologous RBD-binding B cells.

Little is currently known about the cross-reactivity of MBCs elicited by a SARS-CoV-2 infection, and specifically the cross-reactive breadth of RBD-specific MBCs to respond to other VOCs. To this end, we selected 4 D614G-, 4 Alpha-, 4 Beta-, and 4 Delta-infected patients of the cohort, based on the presence of RBD-specific MBCs and availability of material, and evaluated the reactivity of the autologous RBD-specific MBCs to the three other heterologous RBDs. Delta and D614G infections induced the broadest MBC responses, with a substantial proportion of specific total MBCs able to bind three heterologous variants (42.2% and 32.5%, respectively), while 23.3% and 6.9% accounted for autologous RBD-only specific total MBCs, respectively (Fig 4A top panel). In both groups, we observed the highest cross-reactivity to the Alpha RBD (72.4% and 90.6%, respectively). On the contrary, Alpha and Beta infections elicited narrower MBC responses, with a higher proportion of MBCs binding exclusively to the autologous RBD (25.8% and 31.1%, respectively), while the percentage of triple RBD-specific cross-reactive total MBCs accounted for only 14.2% and 20.6%, respectively. As expected from the similarity of RBD amino acid composition, most of the MBC elicited after infection with Alpha cross-reacted with the WT RBD protein (68.7%) and vice versa (90.6%). In the Beta-infected group, we found the highest cross-reactivity towards the Alpha RBD (66.7%), while only 43.0% and 24.6% of the total MBCs recognized WT and Delta RBDs, respectively. On the contrary to the magnitude and phenotype of the MBC responses, the breadth of the total MBC responses did not substantially differ between COVID-19 disease severity (S6A Fig), although group sizes preclude any statistical comparisons. In the classical MBC compartment, we observed similar distributions in terms of B cell breadth among VOC groups, with the broadest response found in Delta-infected patients (43.8% triple cross-reactive MBCs), followed by D614G (39.0%), Beta (16.8%) and Alpha (16.3%) groups (Fig 4A bottom panel and S6B Fig), suggesting that most of the cross-reactive B cells reside within the switched classical MBC subset. To express the cross-reactivity of the total MBC and classical MBC responses as a single value, we calculated the weighted polyfunctionality index, which is a single measure numerically evaluating the degree and variation of polyfunctionality, as described previously [54,55]. The polyfunctionality indices corroborate with both the total MBC and classical MBC results, whereby the Delta and D614G infections having the highest level of polyfunctionality compared to other VOC groups (S7A Fig).

Next, we expanded our cohort by including a second, late-pandemic sample set including Omicron-infected patients to assess cross-binding to the RBD of Omicron BA.1 and BA.2 (Fig 4B top panel). Different D614G- and Delta-infected patients were selected for this second analysis, which showed comparable frequencies of cross-reactive total MBCs binding to the Delta (64.4% vs 61.9%) and D614G RBD (59.1% and 73.9%) between the early- and late pandemic analysis, respectively (S1 Table). Results of three patients, all infected with different VOCs, were excluded for further analysis because they did not meet our criteria of at least ten RBD-specific B cells detected. In total, we included 3 D614G-, 4 Delta-, 3 Omicron BA.1- and 3 Omicron BA.2-infected patients for the late-pandemic analysis. Overall, the Delta and D614G variants again induced the broadest response, with 44.5% and 13.8% triple cross-reactive RBD-specific total MBC, respectively (Figs 4B top panel, and S8A, and S1 Table). In the Delta group, most of the cross-reactivity was observed against the WT RBD (73.9%), although a substantial proportion of B cells also recognized the Omicron BA.1 and BA.2 RBDs (48.2% and 58.0%, respectively). The total MBCs elicited after D614G infection were also able to recognize Omicron BA.1 and Omicron BA.2 RBDs (18.8% and 33.1%, respectively). Consistent with serum neutralization responses [18], primary infection with Omicron BA.1 and BA.2 showed a narrow response, in which 50.0% and 36.4% of B cells exclusively recognized the autologous RBD, while only 5.3% and 3.4% cross-reacted to all three heterologous RBDs (Fig 4B top panel and S1 Table). As expected based on sequence homology in the S protein, the cross-reactivity of B cells of Omicron BA.1-infected patients was higher against the Omicron BA.2 RBD (50.0%), and less against the WT and Delta RBDs (9.2% and 13.2%, respectively). Similarly, Omicron BA.2 infection elicited the strongest cross-reactivity to Omicron BA.1 (59.1%), followed by WT and Delta RBD probes (13.6% and 10.2% of total MBCs, respectively). Remarkably, the percentage of cross-reactive B cells with Omicron BA.2 RBD was highly comparable between the Omicron BA.1 (50.0%) and Delta (58.0%) infected patients. In line with the early-pandemic results, the breadth in the classical MBC subset showed similar results as the total MBC compartments (Figs 4B bottom panel and S8B). In addition, despite not statistically significant, the weighted polyfunctionality indices showed that, for both the total MBC and classical MBC subsets, the Delta infected patients had the highest level of polyfunctionality, while Omicron BA.1 and Omicron BA.2 had the lowest level of polyfunctionality (S7B Fig).

Considering both the early- and late-pandemic datasets, we conclude that SARS-CoV-2 VOCs induce a qualitatively different B cell response in terms of breadth, with the D614G and Delta variants eliciting the broadest MBC responses.

## Discussion

The emergence of multiple antigenically distinct SARS-CoV-2 variants raises concerns regarding the protection provided by natural and vaccine-induced immunity. In this study, we explored the Fc-mediated effector functions of S protein-specific serum antibodies and the RBD-specific B cell responses following a primary SARS-CoV-2 infection with the D614G, Alpha, Beta, Gamma, Delta, Omicron BA.1 or BA.2 variant. We observed a more cross-reactive antibody and B cell response compared to the breadth observed for serum neutralization, with the exception of the limited cross-reactivity of the Alpha-induced B cell response. In addition, between the VOC groups, we observed both quantitative and qualitative differences, which may be partly explained by differences in the immunogenicity of the VOCs.

When studying quantitative differences in the immune responses between VOC groups, we observed the strongest antibody Fc-mediated effector functions and highest frequency of RBD-specific B cell responses in the Alpha-infected patients, followed by the Delta- and D614G-infected patients. All the hospitalized COVID-19 patients of our cohort were infected with one of these three VOCs, demonstrating a strong association between the level of immune response and COVID-19 disease severity. Despite the fact that the association between COVID-19 disease severity and immune activation has been extensively studied [56], there is still ongoing debate about whether this pro-inflammatory state is primarily a consequence of a more severe SARS-CoV-2 infection, and to what extent the immune system actively contributes to the disease severity [57]. We found that the level of FcγR-interaction by S protein-specific serum antibodies was 5 to 6-times stronger in hospitalized compared to non-hospitalized D614G-infected patients, along with a 2.5-times higher frequency of RBD-specific B cells in hospitalized patients. Additionally, hospitalized COVID-19 patients exhibited a higher percentage of double negative B cells, and fewer naive MBCs compared to non-hospitalized patients, which is proposed to represent atypical memory-like B cells, capable of further differentiating into plasma blasts through an extrafollicular maturation pathway [58–61]. To minimize the impact of an uneven distribution of hospitalized COVID-19 patients between VOC groups, we compared VOC groups with comparable COVID-19 disease severity, except when studying the breadth of the RBD-specific B cell compartment. Other study- or patient-specific factors were not statistically significantly correlating with the magnitude of the humoral immune response. When focusing solely on non-hospitalized COVID-19 patients, we found that the Delta-infected patients exhibited an overall superior immune response, showing the highest level of FcγR-interaction by S protein-specific serum antibodies, and the highest frequency of autologous RBD-specific MBCs. In contrast, patients infected with the Omicron sub-variants demonstrated the weakest immune response. A diminished neutralizing antibody response after a primary Omicron infection has been described before [18,20], and is in line with the compromised serologic response observed after an Omicron breakthrough infection in comparison with a Delta breakthrough infection ([3,62]). Moreover, some studies report a reduced immune response following a primary Omicron S protein-based vaccination [63]. Together with similar variations observed in the N protein-specific IgG titers between VOCs, these distinctions among VOC groups suggest differences in the immunogenicity of the SARS-CoV-2 VOCs. These differences in immunogenicity between VOCs are probably multifactorial, including differences in the pathogenicity of VOCs [64,65], affinity of the RBD to ACE2 related to mutations such as N501Y, E484K, and K417N/T in the Alpha, Beta or Gamma variants, differences in the fusogenicity related to mutations such as P681R in the Delta variant [66], or usage of a different entry pathway such as the TMPRSS2-independent entry of Omicron sub-variants [46]. Of note, when comparing the immune response between VOC groups using data of patient with comparable COVID-19 disease severity, we may underestimate differences in immunogenicity by correcting for VOC pathogenicity.

These variations in the magnitude of FcγR-interaction by S protein-specific antibodies, and RBD-specific B cell responses strongly correlated with S protein-specific IgG binding titers. More specifically, the autologous S protein-specific IgG response very strongly correlated with the level of interaction with FcγRIIa and FcγRIIIa. In addition, we only found minor differences in the ratio of FcγRIIa and FcγRIIIa-interaction between the VOC groups. This suggests that the proportion of S protein-specific antibodies with Fc-mediated effector functions are preserved among the VOCs, and none of the variants induce a lower of higher proportion of antibodies mediating ADCP or ADCC compared to other VOCs. The finding that these correlations did not substantially differ between the autologous and heterologous S protein-specific responses indicates that, irrespective of whether antibodies target the highly variable RBD or more conserved regions of the S protein, these antibodies interact with the FcγRIIa and FcγRIIIa with the same capacity in our assay. In addition, our results align with what has been previously described by Richardson *et al*., who showed minor differences in breadth of ADCP and ADCC for D614G and Beta-infected patients when considering S protein-specific IgG titers [40]. The most significant difference was found for the antibody-dependent complement deposition (ADCD), which was outside the scope of this study.

To further investigate the relationship between S protein-specific IgG titers and Fc-mediated effector functions, we measured the S protein-specific IgG3 and IgG1 responses, both known for their strong affinity to FcγRs [49]. The correlations between FcγR-binding and S protein-specific IgG subclass levels found, were similar for IgG3 and IgG1, indicating that both subclasses may stimulate innate immune cells to the same degree. However, given that IgG1 is more abundant in serum, IgG1 is likely the primary driver of FcγR-engagement and Fc-mediated effector functions following SARS-CoV-2 infection. This correlation between S protein-specific IgG and FcγR-interaction has also been observed following COVID-19 vaccination [39]. Interestingly, the Alpha-infected patients exhibited a relatively low IgG3 responses compared to other VOC groups, despite the large proportion of hospitalized COVID-19 patients in the Alpha group, and the fact that higher IgG3 titers have been reported in more severely ill COVID-19 patients [67]. This may be explained by the low number of ICU admitted patients in our cohort (one D614G- and one Alpha-infected patient).

Regarding the RBD-specific B cell responses, aside from the quantitative differences between the VOC groups mentioned earlier, we also observed variations in the quality of the response among the VOCs causing the infection. Beta-infected patients showed a more immature antigen-specific B cell response with higher levels of naive B cells and a predominant IgM isotype. In contrast, Delta-infected patients exhibited a more mature response with high frequencies of classical MBCs and an IgG isotype. These differences in the quality of the B cell responses among VOC groups are unlikely to be caused by sampling timing variation, given the minimal variability in sampling timing and the weak correlation between days post symptom onset and MBC isotype. Interestingly, the Omicron BA.2-infected patients showed a relatively high proportion of IgG^-^IgM^-^ RBD-specific MBCs, suggesting that these MBCs might have class-switched to IgA. IgA is an important isotype for mucosal protective immunity. The possible presence of relatively high level of IgA^+^ RBD-specific MBCs in Omicron BA.2-infected patients is in line with Omicron having shifted its tropism away from the lower respiratory tract and causing often milder upper respiratory tract COVID-19 disease [64,68].

Because of the emergence of antigenically distinct variants and the waning of serum antibodies overtime, we then studied the breadth of antibodies mediating Fc-effector functions and the RBD-specific MBC response in comparison with the previously determined serum neutralization responses [18]. The Fc-mediated effector functions mediated by S protein-specific antibodies were broader than serum neutralization, likely because these antibodies can target epitopes on the entire surface of the S protein, including more conserved epitopes outside the RBD. This is in accordance with the observed preservation of interaction with FcγRIIa and FcγRIIIa of Omicron S protein-specific antibodies following COVID-19 vaccination [39]. For the RBD-specific MBC responses, the serum neutralization did not always mirror the cross-reactivity of the MBC response. Consistent with the neutralization responses, we observed a highly cross-reactive MBC response elicited by the D614G variant. In addition, for the Omicron sub-variants, the weak and primarily autologous strain-focused neutralization was mirrored by only 3.4% and 5.3% of MBCs being triple cross-reactive following an Omicron BA.1 and BA.2 infection, respectively. However, discrepancies between the cross-neutralization and cross-reactivity of MBC response were found, especially for the Delta and Alpha variant. The Delta variant elicited a broadly reactive MBC response with 42.2% and 44.5% MBCs being reactive against all four RBDs used in the early and late pandemic analysis, respectively. In addition, Delta-infected patients stood out in the frequencies of total MBCs reactive against Omicron BA.1 (48.2%) and BA.2 RBD (58%). These frequencies were comparable for the cross-reactivity between the Omicron RBDs in the Omicron BA.1- (50.0%) and BA.2- (59.1%) infected patients. This breadth starkly contrasts the narrow and autologous strain-specific neutralization previously found in sera of these same Delta-infected patients [18]. On the other hand, the Alpha variant exhibited a very narrow MBC response, despite the fact that it induced the broadest neutralizing antibody responses and having the highest level of interaction with FcγRs. Other than for the antibody Fc-mediated effector functions, these differences cannot primarily be explained by the availability of conserved epitopes, as the majority of neutralizing antibodies target the RBD. Hence, further research is needed to characterize the neutralizing capacity of RBD-specific MBCs. One possible explanation for the discrepancy in cross-reactivity between MBCs and serum antibodies could be that this study only focused on RBD-specific MBCs, while another important antigenic site of the S protein is the NTD. As the NTD is also targeted by many potent neutralizing antibodies, the breadth of the MBC responses in this study may be underestimated. Notably, the extent to which these MBCs will indeed be reactivated upon re-exposure to an antigenically distinct variant remains a subject of debate, as *de novo* responses may also have a substantial contribution to the immune response following re-exposure [45,69].

While our primary VOC cohort is unique, the main limitation of this study is the low number of primary SARS-CoV-2 infected patients per VOC group, especially in the Gamma- and Omicron BA.2-infected groups. These low numbers are primarily due to the Beta and Gamma variants never being endemic in the Netherlands, the general high reproductive rate of SARS-CoV-2 that reduces the number of primary infections preceding the emergence of the Omicron sub-variants, and the high vaccination coverage rate in the Netherlands, which further limited the occurrence of primary Omicron infections. Despite these relatively low numbers, we showed that VOCs are not only antigenically different, but also found differences in immunogenicity. Further research is needed to pinpoint which mutations are primarily responsible for these differences, which can in turn further guide the design of COVID-19 vaccines towards the induction of broadly protective immunity against antigenically distinct variants.

Lastly, the preserved cross-reactivity of S-protein specific antibodies with Fc-mediated effector functions and the discrepancies between the breadth of the MBC compartment and neutralization, underlines the importance of studying the humoral immune system beyond serum neutralization to get a better understanding on the degree of protection against newly emerging variants of concern.

## Materials and methods

### Ethics statement

The COSCA and RECoVERED studies were conducted at the Amsterdam University Medical Centers, the Netherlands, and approved by the local ethical committee (METC Amsterdam UMC, NL73281.018.20 and NL73759.018.20 respectively). All individuals provided written informed consent before participating in the study.

### Study population

Primary PCR-confirmed SARS-CoV-2 infected adults (n = 67) without any history of COVID-19 vaccination from a previously described cohort were included in this study [18,47]. All patients had either a sequence confirmed VOC infection, or met our previously defined criteria of a high likelihood of a VOC infection, except one Alpha-infected patient. This patient (COSCA-304) was a household member of one of the sequence-confirmed Alpha-infected patients. Serum neutralization of this patient showed an Alpha VOC-specific neutralization pattern. This patient was included to better balance the number of non-hospitalized and hospitalized Alpha-infected patients in our cohort. As negative control we used sera of n = 11 pre-pandemic healthy donors, and n = 2 plasma pools of pre-pandemic healthy donors, both obtained via Sanquin Research, Amsterdam, The Netherlands.

### Soluble protein design, production and purification

Soluble, pre-fusion stabilized homotrimeric SARS-CoV-2 S proteins with a T4 trimerization domain and an hexahistidine (His) tag were generated, as described previously [70]. In short, the WT SARS-CoV-2 S sequence (Wuhan Hu-1; GenBank: MN908947.3) was used as template and mutations were inserted to produce the Alpha, Beta, Gamma, Delta and Omicron BA.1 S proteins, as described previously [6]. Compared to the WT SARS-CoV-2 S protein, the Omicron BA.2 S protein harbored the following mutations: T19I, L24S, Δ25/27, G142D, V213G, G339D, S371F, S373P, S375F, T376A, D405N, R408S, K417N, N440K, S447N, T478K, E484A, Q493R, Q498R, N501Y, Y505H, D614G, H655Y, N679K, P681H, N764K, D796Y, Q954H, N969K. SARS-CoV-2 N protein was kindly provided by Gestur Vidarsson, Sanquin Research, Amsterdam, The Netherlands.

The design of pre-fusion stabilized trimeric RSV-F glycoprotein (Strain A1) was described previously [71], as was the design of the trimeric HA of influenza virus (H1N1pdm2009 A/Netherlands/602/2009) [72]. Both were produced in human embryonic kidney (HEK)293F cells maintained in FreeStyle medium (Life Technologies), and purified by Ni-NTA chromatography followed by size exclusion chromatography, as described previously [47].

In addition to full-length S proteins, soluble RBD proteins of the WT, Alpha, Beta, Delta, Omicron BA.1 and BA.2 variants were ordered as gBlock gene fragments (Integrated DNA Technologies), cloned in a pPPI4 expression vector [73] containing an His-Tag and/or an Avi-Tag with Gibson Assembly (ThermoFisher). After verification by Sanger sequencing, all soluble RBD proteins were produced in HEK293F cells (Invitrogen), as previously described [70], and purified using Ni-NTA columns. After purification, Avi-tagged RBD proteins were biotinylated with a BirA500 biotin-ligase reaction kit according to the manufacturer’s instruction (Avidity).

High affinity allelic variants of human FcγRIIa-H131 and FcγRIIIa-V158 ectodomain dimers were produced in HEK293F cells (Invitrogen), as described previously [48]. Plasmids were designed by Bruce Wines and Mark Hogarth of the Burnet Institute in Melbourne, Australia and contained the genes followed by an His-Tag and Avi-Tag [46]. The FcγRIIa-H131 and FcγRIIIa-V158 ectodomains were biotinylated as described above. All proteins were further purified using size exclusion chromatography and stored at -80°C until use.

### Luminex assay to measure serum IgG binding and the level of interaction with FcγRs

SARS-CoV-2 S proteins were covalently coupled to Magplex beads (Luminex Corporation) in a ratio of 75 μg S protein to 12.5 million beads, as previously described [47]. Other proteins were coupled in an equimolar amount compared to the S proteins. To prevent non-specific binding, coupled beads were blocked by incubating them for 30 min with phosphate-buffered saline (PBS) containing 2% bovine serum albumin (BSA), 3% fetal calf serum (FCS) and 0.02% Tween-20 at pH 7.0. Thereafter, beads were stored in the dark at 4°C in PBS containing 0.05% sodium azide and used within one year after coupling. 50 μL of a bead mixture containing all different SARS-CoV-2 VOC S proteins, the N protein and the respiratory control proteins in a concentration of 20 beads per μL were added to 50 μL of diluted serum and incubated overnight on a rotator at 4°C, as described previously [47,48]. Corresponding with the optimal dilution found in our pilot studies, sera was diluted 1:10,000 for measuring IgG levels, 1:10,000 and 1:1,000 for IgG1 levels, and 1:500 and 1:5,000 for IgG3 levels. For IgG binding against the respiratory control proteins and N protein, sera were diluted 1:100,000. For binding to FcγRIIa, FcγRIIIa, multiple dilutions were used to find the optimal serum dilution per sample ranging from 1:50 to 1:50,000. Serum was incubated with the bead mixture overnight at 4°C on a rotator. The next day plates were washed twice with Tris-buffered saline (TBS) containing 0.05% Tween-20 (TBST) and resuspended with 50 μL (1.3 μg/mL) goat-anti-human IgG-PE (Southern Biotech), mouse-anti-human IgG3-PE (Southern Biotech) or the FcγR ectodomains and incubated for 2 h on a plate shaker at room temperature (RT). For the FcγR binding, plates were washed afterwards and incubated with 50 μL Streptavidin-PE (Invitrogen) for 1 h on a plate shaker at RT. Next, plates were washed twice with TBST before adding 70 μL of Magpix drive fluid (Luminex). Read-out was performed on a Magpix (Luminex). Antibody binding was expressed as the Median Fluorescence Intensity (MFI) of approximately 50 to 150 beads per well and corrected for background signals by subtracting the MFI of wells containing only buffer and beads. S protein- and N protein-specific IgG binding expressed as MFI were converted into binding antibody units per mL (BAU/mL) using the WHO International Standard for anti-SARS-CoV-2 immunoglobulin (NIBSC 20/136) [74]. Binding levels of IgG3 or the level of interaction with FcγRs were corrected for the dilution used, and thereafter expressed as Arbitrary Units (AU/mL).

### ADCC assay

In this ADCC assay, antibody-dependent NK cell activation and degranulation is used as surrogate for cellular cytotoxicity [75]. First, half-area ELISA microplates (Maxisorb) were coated overnight at 4°C with 2 μg/mL SARS-CoV-2 WT S proteins. The next day plates were blocked with 1% BSA in PBS. After washing plates with TBS, WT S protein-coated plates were incubated with 50 μL of serum of a subset of SARS-CoV-2 infected patients for 1 h at 37°C. A serum dilution of 1:20 with blocking buffer (PBS-1% BSA) was chosen after titration of sera in a pilot experiment. One of the serum pool from pre-pandemic healthy donors was used as negative control and 5 μg/mL of recombinant, unmodified and afucosylated COVA1-18 monoclonal antibodies [38] as positive controls. NK cells were derived from PBMCs from healthy donors obtained from Sanquin, as described previously [76]. In short, NK cells were enriched by positive selection from human PBMCs using Miltenyi’s CD56 MicroBeads following manufacturer’s protocol and stimulated overnight at 37°C with 10 ng/mL IL-15 in Iscove’s Modified Dulbecco’s Medium supplemented with 10% FCS and 100 U/mL penicillin/streptomycin. NK cells were added onto the S protein coated ELISA plates at 50,000 cells per well and incubated for 3 h at 37°C in the presence of 10 ng/mL IL-15 (Invitrogen) and 5 μg/mL BrefeldynA (Biolegend). After incubation, NK cells were transferred into a 96 well V-bottom microplate and stained with anti-CD16 BV421 (Clone 3G8; Biolegend), anti-CD107 APC (Clone H4A3; Biolegend) and fixable viability dye ef780 (Invitrogen 1:5000). After incubating cells in a staining solution for 1 h at 4°C, cells were washed twice with FACS buffer (2% FCS in PBS), and subsequently fixed with the Fixation/Permeabilization Kit (BD biosciences). After fixation and permeabilization, NK cells were stained intracellularly with anti-IFNγ PE (Clone B27; Biolegend) dissolved in Wash/Perm buffer. After intracellular staining, cells were washed twice in the wash buffer, once in the FACS buffer and then resuspended in the FACS buffer for flow cytometry analysis. NK activation was determined by using a BD FACSymphony A1 Cell Analyzer and analyzed with FlowJo software. The percentage of double positive (CD107^+^, IFNγ^+^) NK cells were used to compare with the FcγRIIIa binding data obtained using the Luminex assay. 33% of the COVID-19 infected patients were tested twice in the ADCC assay and results were consistent between the two experiments.

### ADCP assay

In the ADCP assay, we tested the activation of THP-1 effector cells by opsonized Fluorescent Neutravidin beads (Invitrogen) coated with SARS-CoV-2 WT S protein, as described previously [76]. In short, biotinylated SARS-CoV-2 WT S protein was incubated overnight at 4°C with Neutravidin beads in PBS (5 μg/10 μL beads). After washing the beads twice with 2% BSA in PBS and resuspending them in 2% BSA in PBS, 50 μL of serum of a subset of SARS-CoV-2 infected patients was added to 900,000 beads and incubated for 2 h at 37°C. A 3-fold serum dilution starting at 1:3,000 was chosen after titration of sera in a pilot experiment. After incubation, the plates were washed three times with PBS 2% FCS, followed by adding 5x10^6^ THP-1 effector cells. The plates were then quickly spun down to promote beads-to-cell contact and incubated for 5 h at 37°C. After incubation, the cells were washed and stained with a fixable viability dye (ef780, Biolegend) for 30 minutes at 4°C. Subsequently, the cells were washed twice with PBS 2% FCS, resuspended in PBS 2% FCS and ADCP was measured on the BD FACSymphony A1 Cell Analyzer and analyzed with FlowJo software (BD Biosciences). Phagocytic activity was expressed as the area under the curve (AUC) of the MFI. The ADCP AUC was compared with the level of FcγRIIa ectodomain dimer-interaction obtained using the Luminex assay. 79% of the COVID-19 infected patients were tested twice in the ADCP assay and results were consistent between the two experiments.

### Flow cytometry analysis of RBD-specific B cells

Biotinylated RBD antigens of the WT, Alpha, Beta, Delta, Omicron BA.1, and BA.2 variants were individually multimerized with fluorescently-labeled streptavidin (BB515, BD Biosciences; AF647, Biolegend; BUV615, Biolegend; PE-Cy7, BD Biosciences; BV421, Biolegend). Briefly, biotinylated proteins and fluorescently-labeled streptavidin were mixed and incubated at 4°C for 1 h at a 2:1 molar ratio of protein to streptavidin conjugates. 10mM biotin (Genecopoiea) was used to quench unbound streptavidin conjugates for at least 15 min. Frozen PBMC samples were stained with the antigen probe mix and live/DEAD dye together with extracellular antibody markers for 30 min at 4°C (S2 Table). Stained samples were subsequently washed twice with FACS buffer (PBS supplemented with 1 mM EDTA and 2% FCS) and acquired on the BD LSRFortessa for cell analysis. Analysis was performed by using FlowJo software (BD Biosciences). To study the cross-reactivity of MBCs induced by the different VOCs, we used Simplified Presentation of Incredibly Complex Evaluations data software version 6 (SPICE6) [77]. COSCA samples 026, 360, 361 contained less than 10 total RBD-specific B cells and were excluded from the cross-reactivity analysis. We calculated the polyfunctionality index, which is a single measure numerically evaluating the degree and variation of polyfunctionality, as described previously [54,55]. In this study, the parameter q was set to 1 and not iteratively optimized. Since polyfunctional responses among VOCs with closer genetic differences based on antigenic cartography distances are likely to be more common, we calculated the polyfunctionality index such that responses with further genetic distances were weighted more heavily. This was accomplished by dividing the genetic distances of the involved polyfunctional responses by the total of all genetic distances included in the index. Distances between VOCs were previously determined by using an antigenic cartography [18].

### Statistical analysis

For the Luminex analysis a Lower Limit of Quantification (LLQ) was set at 10 MFI as a higher degree of inter-assay variability was observed below this value. The corresponding LLQ was 2.2 BAU/mL for the IgG binding, 21 AU/mL for IgG3 and IgG1 binding and 0.77 and 0.43 AU/mL for the level of interaction with FcγRIIa and FcγRIIIa, respectively. Values below these LLQ were substituted with the LLQ divided by the square root of 2 prior to analysis [78]. To reduce the bias from outlying values, we calculated the geometric mean titer of the humoral responses per VOC group to best take into account outliers. The large number of zero values in the B cell analysis precluded calculation of the geometric mean titer, and median values were presented for this analysis instead. The Kruskal-Wallis test was used as an overall test for differences across groups with comparable COVID-19 disease severity (i.e. hospitalized versus non-hospitalized). Pairwise comparisons between VOCs were conducted *post hoc* using the Mann-Whitney U test (for non-paired data) or the Wilcoxon signed-rank test (for paired data). P-values were corrected for multiple comparisons using the Benjamini-Hochberg procedure. A q-value <0.05 was deemed statistically significant.

Given the small sample sizes and sparse data from some VOC, we used a penalized regression approach whereby uncertain estimates from the data are pulled towards more realistic ones with the use of prior distributions. We estimated the mean frequency B cells and their 95% credible intervals (CrI) using a Bayesian linear model. A weakly-informative prior was specified for all model parameters as a uniform distribution between -10,000 and 10,000. Using these priors together with the data, a posterior distribution of the means was estimated with Markov Chain Monte Carlo methods from the “bayes” family of commands in STATA. The median of this distribution defined the estimated mean level and the 2.5% and 97.5% quantiles defined the 95% credible interval (CrI). These models were used to determine if differences remained while accounting for sparse data.

GraphPad Prism 9 was used for the statistical analysis and the creation of graphs. Flowjo version 10.9 was used for flow cytometry analysis, including the B cell, ADCC and ADCP analysis. Cross-reactivity of MBCs were analyzed using Simplified Presentation of Incredibly Complex Evaluations data software version 6 (SPICE6) [77].

## Supporting information

S1 Table Cross-reactivity of RBD-specific total and classical MBCs against VOCs. On the left, the frequencies of RBD-specific MBCs against the D614G, Alpha, Beta and Delta RBD are listed for the early-pandemic dataset. On the right, the frequencies of RBD-specific MBCs against the D614G, Delta, Omicron BA.1, and Omicron BA.2 are listed for the late-pandemic dataset.
(XLSX)

S2 Table Staining of the RBD-probes and the extracellular antibody markers. (XLSX)

S1 Fig S protein-specific serum responses. A) Level of interaction of S protein-specific serum antibodies with FcγRIIa (left panel) and FcγRIIIa ectodomain dimers (right panel), expressed as arbitrary units per mL (AU/mL). Convalescent sera were grouped by VOC causing the infection and separated based on COVID-19 disease severity. Dots represent the posterior mean level of interaction as calculated from the Bayesian linear regression model. The bars represent the 95% credible interval of the posterior mean. Circles and triangles indicate non-hospitalized and hospitalized individuals, respectively. B) Linear regression analysis between the level of interaction with FcγRIIIa and the S protein-specific IgG titers in binding antibody units per mL (BAU/mL). The gray bar indicates the lower limit of quantification. C) Spearman correlation between the days post symptom onset (DPSO) and S protein-specific IgG titers in binding antibody units per mL (BAU/mL). The gray bar indicates the lower limit of quantification. D) Serum IgG response to the fusion protein of Respiratory Syncytial Virus (RSV-F) (left panel) and hemagglutinin (HA) of influenza virus (right panel). Convalescent sera is grouped by VOC causing the infection. Kruskal-Wallis test is used to test for differences between groups. E) Spearman correlation between age and IgG binding to RSV-F (left panel) and HA (right panel). F) Nucleocapsid (N) protein-specific antibody binding of convalescent sera. Sera is plotted and analyzed in the same manner as Fig 1A. G) S protein-specific IgG titers in BAU/mL calculated using Bayesian Statistics. H) Linear regression analysis between the level of interaction with FcγRIIIa and S protein-specific IgG3 titers in AU/mL. I) Linear regression analysis between S protein-specific IgG1 titers in AU/mL and the level of interaction with FcγRIIa (left panel) and FcγRIIIa (right panel) in AU/mL. J) Ratio between S protein-specific IgG3 subclass and total S protein-specific IgG titers. A Mann-Whitney U test with Benjamini-Hochberg correction is used to compare titers between the VOC groups. Only statistically significant differences are reported (* = q<0.05, ** = q<0.01).
(TIF)

S2 Fig Cross-reactivity of S protein-binding serum responses with FcγR-interaction. The level of interaction with FcγRIIa (A) and FcγRIIIa (B) by autologous and heterologous S protein-specific serum antibodies. Patients are grouped by VOC causing the infection. Autologous S protein binding is highlighted using a light blue bar. Geometric mean binding titers are highlighted as squares. The background shows the individual binding levels: triangles indicate hospitalized patients, dots non-hospitalized patients. The gray bar indicates the limit of quantification (LOQ) of the assay. The Wilcoxon test with Benjamini-Hochberg correction is used to test differences in level of interaction with FcγRs by autologous versus heterologous S protein-specific antibodies. Only statistically significant differences are reported (*q<0.05, **q<0.01, ***q<0.001). C) The ratio of FcγRIIa and FcγRIIIa-interaction by autologous and heterologous S protein-binding antibodies. D) Linear regression slopes of the level of interaction with FcγRIIa (left panel) and FcγRIIIa (right panel) with corresponding autologous and heterologous S protein-specific IgG titers.
(TIF)

S3 Fig Correlation between the level of interaction with FcγRs with corresponding S protein-specific IgG titers. Spearman correlations between level of interaction with FcγRIIa (A) and FcγRIIIa (B) with corresponding S protein-specific IgG titers in binding antibody units per mL (BAU/mL).
(TIF)

S4 Fig Characterization of autologous RBD-specific B cells using flow cytometry. A) Representative gating strategy used to identify RBD-specific B cells. For each VOC group, the autologous RBD was differentially labeled with two fluorochromes (AF647 and BV421), as in the reported example. RBD-specific B cells were then analyzed according to surface marker expression (IgD, CD27, IgG and IgM). SSC-A, side scatter area; FSC-H, forward scatter height; FSC-A, forward scatter area. RBD, receptor binding domain; MBCs, memory B cells. B) Differences in magnitude of autologous RBD-specific B cell responses between non-hospitalized and hospitalized patients in the total B cell (left panel) and classical MBC (right panel) compartment. Mann-Whitney U test (*p<0.05, **p<0.01). Bars show median values for each group. C) Frequency of RBD-specific total B cell (left), total MBC (middle), and classical MBC compartment (right panel) using Bayesian Statistics. D) Spearman correlation between percentage of autologous RBD-specific classical MBCs and serum pseudovirus neutralization titers (IU/mL). E) Phenotype of CD19^+^ autologous RBD-specific B cells, divided by hospitalization status. NSM, non-switched memory; SM, switched memory, DN, double negative.
(TIF)

S5 Fig Characterization of the phenotype of autologous RBD-specific B cells using flow cytometry. A) From left to right: the phenotype of the RBD-specific classical memory B cell (MBC), naïve B cell, non-switched MBC and double negative B cell compartments using Bayesian statistics. B) Isotyping of total CD19^+^ autologous RBD-specific B cells, divided by VOC causing the infection, and COVID-19 disease severity. C) Isotyping of RBD-specific classical, switched MBCs, divided by VOC causing the infection, and COVID-19 severity. D) Spearman correlation between autologous serum neutralization titers and percentage of autologous RBD-specific IgG^+^ (left panel) and IgM^+^ B cells (right panel). E) Spearman correlation between days post symptom onset (DPSO) and % RBD-specific IgG (left panel), % RBD-specific IgM (middle panel) and % RBD-specific total MBCs (right panel).
(TIF)

S6 Fig Individual plots of the breadth of RBD-specific MBCs of the early-pandemic dataset. The cross-reactivity legend indicates total MBCs (A) or classical MBCs (B) that recognize the autologous RBD only (0), or bind one, two or three other heterologous RBDs. Patients highlighted in red have been hospitalized.
(TIF)

S7 Fig Weighted polyfunctionality indices of RBD-specific B cells. Weighted polyfunctionality indices for the early-pandemic (A) or late-pandemic dataset (B) for the RBD-specific total B cell compartment (left) and classical MBC compartment (right). The indices are calculated taking into account the antigenic distances between the VOCs [18], and compared using a Mann-Whitney U test.
(TIF)

S8 Fig Individual plots of the breadth of RBD-specific MBCs of the late-pandemic dataset. The cross-reactivity legend indicates total MBCs (A) or classical MBCs (B) that recognize the autologous RBD only (0), or bind one, two or three other heterologous RBDs. None of the patients included in this analysis were hospitalized.
(TIF)
