# Supplementary figures and images for: Primary SARS-CoV-2 variant of concern infections elicit broad antibody Fc-mediated effector functions and memory B cell responses
Source: PLoS Pathog. 2024 Aug 15;20(8):e1012453. doi: 10.1371/journal.ppat.1012453 (PMC11349224; doi:10.1371/journal.ppat.1012453)

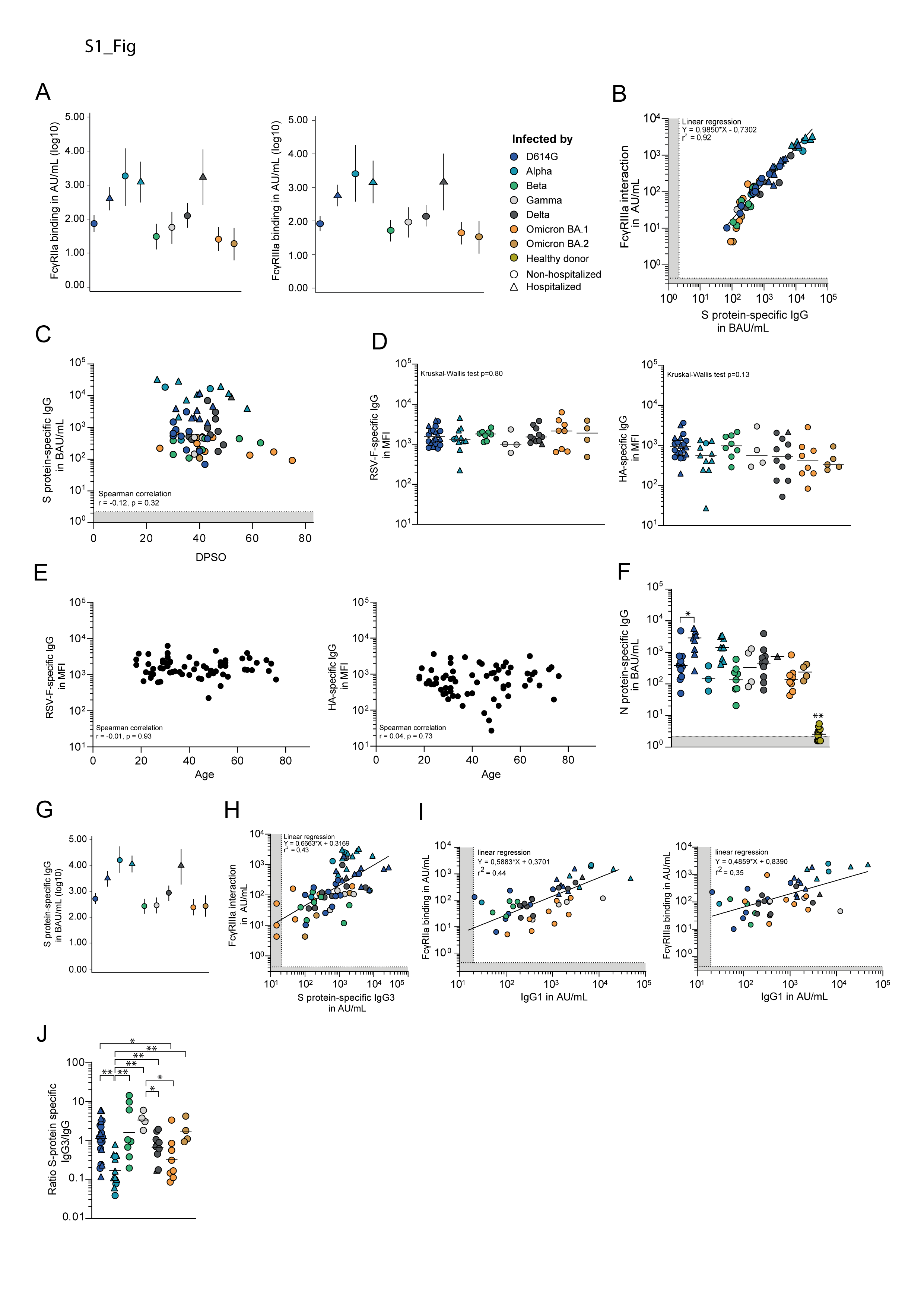

Supplement: S1 Fig — A) Level of interaction of S protein-specific serum antibodies with FcγRIIa (left panel) and FcγRIIIa ectodomain dimers (right panel), expressed as arbitrary units per mL (AU/mL). Convalescent sera were grouped by VOC causing the infection and separated based on COVID-19 disease severity. Dots represent the posterior mean level of interaction as calculated from the Bayesian linear regression model. The bars represent the 95% credible interval of the posterior mean. Circles and triangles indicate non-hospitalized and hospitalized individuals, respectively. B) Linear regression analysis between the level of interaction with FcγRIIIa and the S protein-specific IgG titers in binding antibody units per mL (BAU/mL). The gray bar indicates the lower limit of quantification. C) Spearman correlation between the days post symptom onset (DPSO) and S protein-specific IgG titers in binding antibody units per mL (BAU/mL). The gray bar indicates the lower limit of quantification. D) Serum IgG response to the fusion protein of Respiratory Syncytial Virus (RSV-F) (left panel) and hemagglutinin (HA) of influenza virus (right panel). Convalescent sera is grouped by VOC causing the infection. Kruskal-Wallis test is used to test for differences between groups. E) Spearman correlation between age and IgG binding to RSV-F (left panel) and HA (right panel). F) Nucleocapsid (N) protein-specific antibody binding of convalescent sera. Sera is plotted and analyzed in the same manner as Fig 1A. G) S protein-specific IgG titers in BAU/mL calculated using Bayesian Statistics. H) Linear regression analysis between the level of interaction with FcγRIIIa and S protein-specific IgG3 titers in AU/mL. I) Linear regression analysis between S protein-specific IgG1 titers in AU/mL and the level of interaction with FcγRIIa (left panel) and FcγRIIIa (right panel) in AU/mL. J) Ratio between S protein-specific IgG3 subclass and total S protein-specific IgG titers. A Mann-Whitney U test with Benjamini-Hochberg [file ppat.1012453.s003.tif]

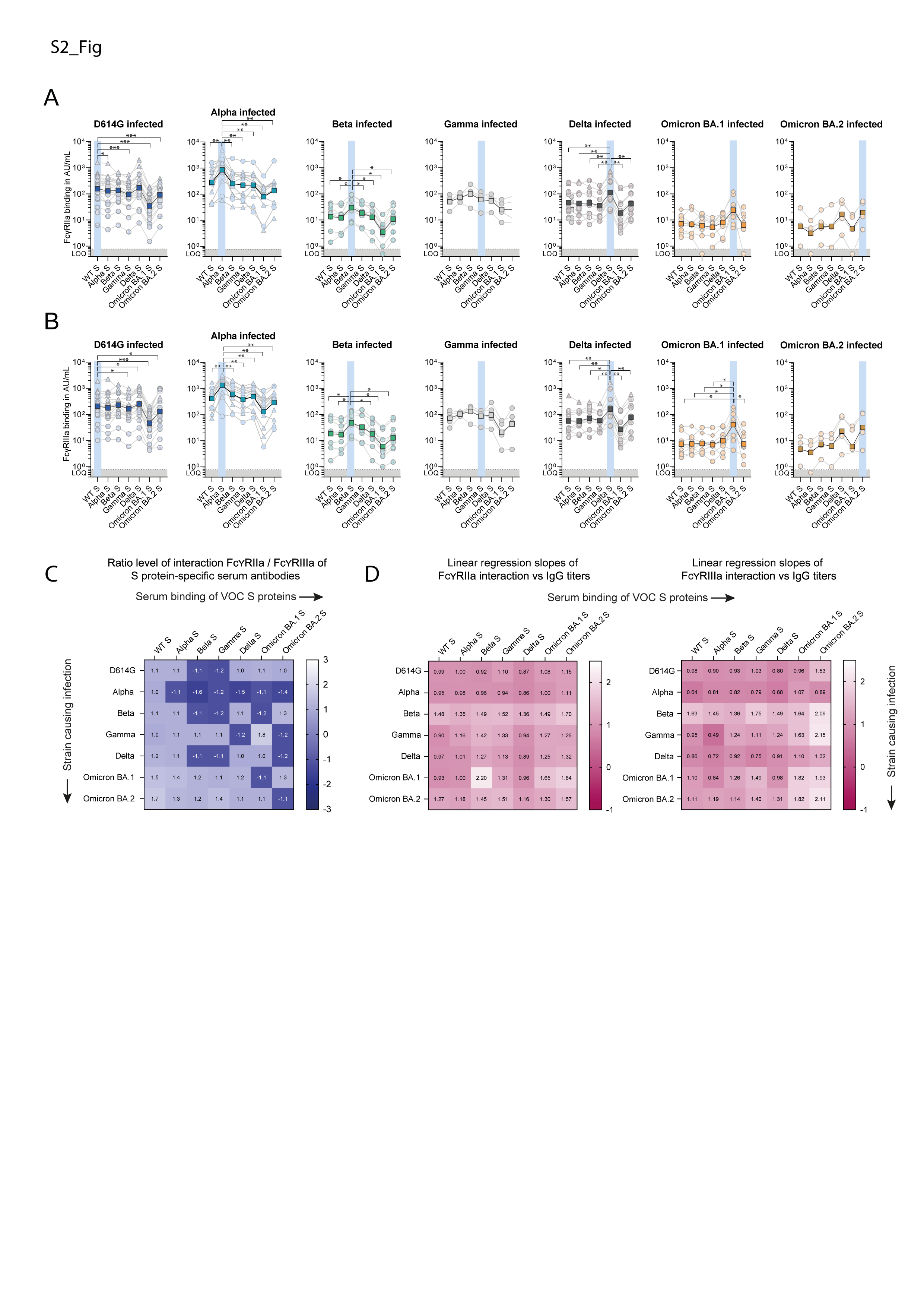

Supplement: S2 Fig — The level of interaction with FcγRIIa (A) and FcγRIIIa (B) by autologous and heterologous S protein-specific serum antibodies. Patients are grouped by VOC causing the infection. Autologous S protein binding is highlighted using a light blue bar. Geometric mean binding titers are highlighted as squares. The background shows the individual binding levels: triangles indicate hospitalized patients, dots non-hospitalized patients. The gray bar indicates the limit of quantification (LOQ) of the assay. The Wilcoxon test with Benjamini-Hochberg correction is used to test differences in level of interaction with FcγRs by autologous versus heterologous S protein-specific antibodies. Only statistically significant differences are reported (*q<0.05, **q<0.01, ***q<0.001). C) The ratio of FcγRIIa and FcγRIIIa-interaction by autologous and heterologous S protein-binding antibodies. D) Linear regression slopes of the level of interaction with FcγRIIa (left panel) and FcγRIIIa (right panel) with corresponding autologous and heterologous S protein-specific IgG titers. (TIF) [file ppat.1012453.s004.tif]

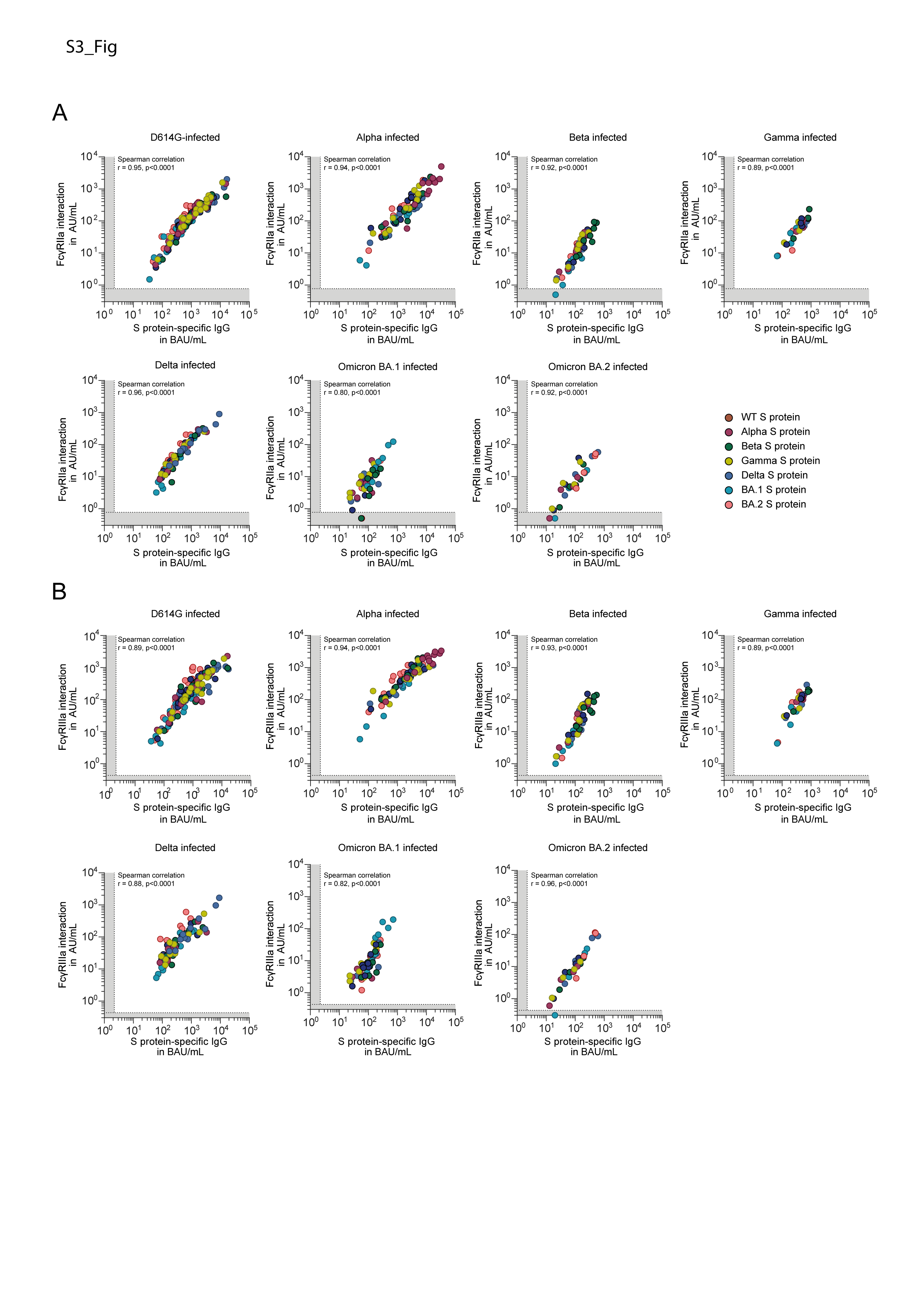

Supplement: S3 Fig — Spearman correlations between level of interaction with FcγRIIa (A) and FcγRIIIa (B) with corresponding S protein-specific IgG titers in binding antibody units per mL (BAU/mL). (TIF) [file ppat.1012453.s005.tif]

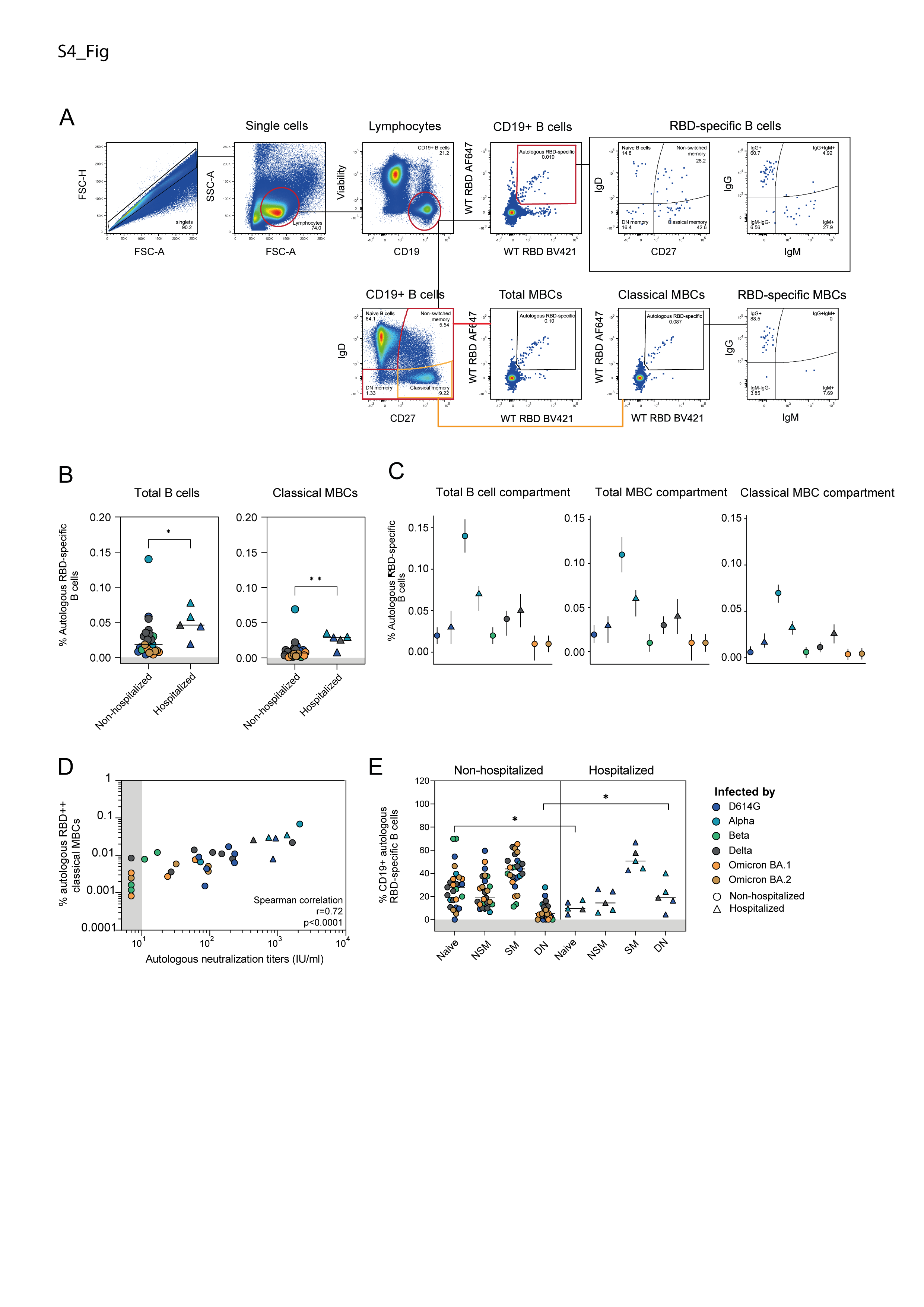

Supplement: S4 Fig — A) Representative gating strategy used to identify RBD-specific B cells. For each VOC group, the autologous RBD was differentially labeled with two fluorochromes (AF647 and BV421), as in the reported example. RBD-specific B cells were then analyzed according to surface marker expression (IgD, CD27, IgG and IgM). SSC-A, side scatter area; FSC-H, forward scatter height; FSC-A, forward scatter area. RBD, receptor binding domain; MBCs, memory B cells. B) Differences in magnitude of autologous RBD-specific B cell responses between non-hospitalized and hospitalized patients in the total B cell (left panel) and classical MBC (right panel) compartment. Mann-Whitney U test (*p<0.05, **p<0.01). Bars show median values for each group. C) Frequency of RBD-specific total B cell (left), total MBC (middle), and classical MBC compartment (right panel) using Bayesian Statistics. D) Spearman correlation between percentage of autologous RBD-specific classical MBCs and serum pseudovirus neutralization titers (IU/mL). E) Phenotype of CD19+ autologous RBD-specific B cells, divided by hospitalization status. NSM, non-switched memory; SM, switched memory, DN, double negative. (TIF) [file ppat.1012453.s006.tif]

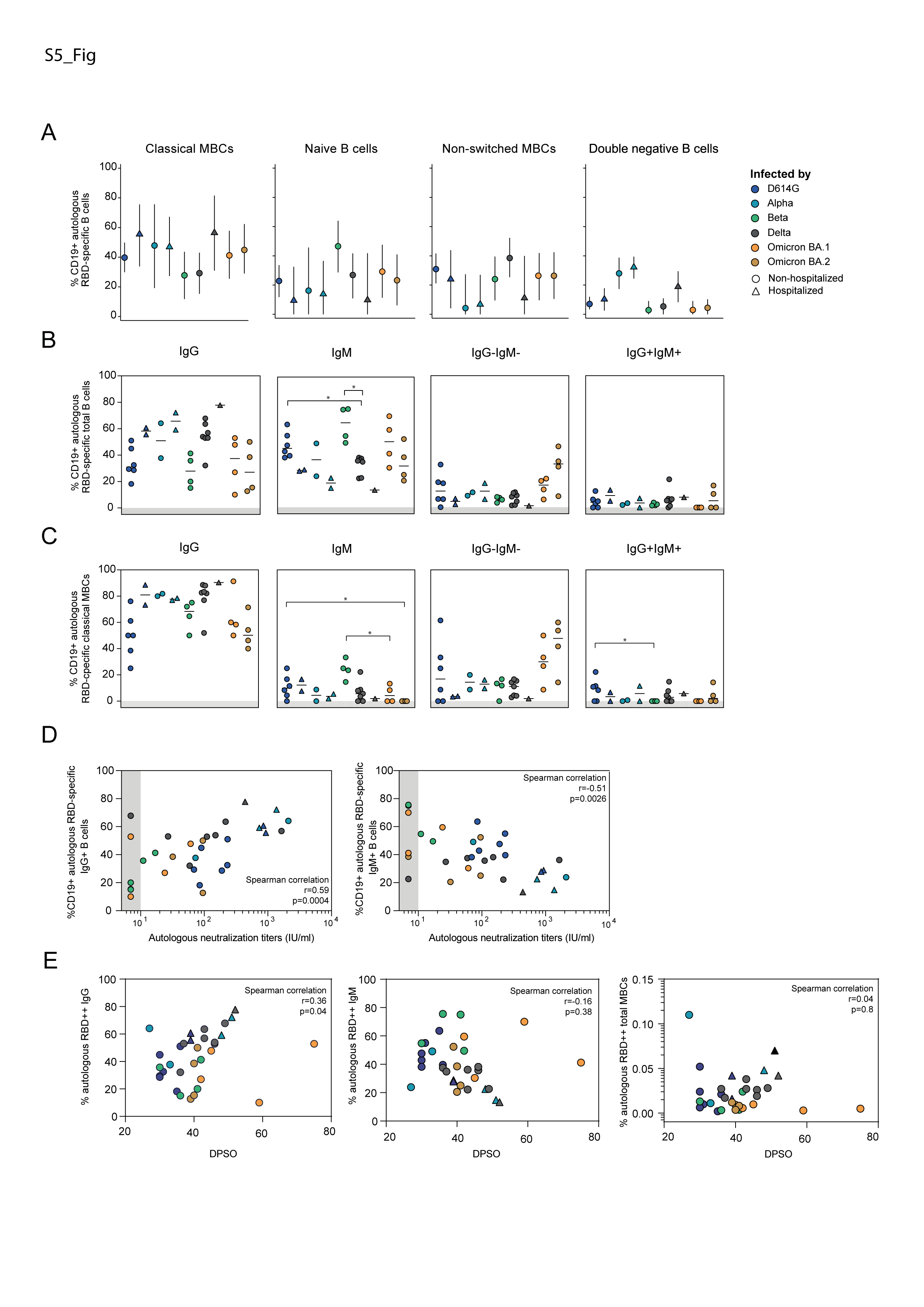

Supplement: S5 Fig — A) From left to right: the phenotype of the RBD-specific classical memory B cell (MBC), naïve B cell, non-switched MBC and double negative B cell compartments using Bayesian statistics. B) Isotyping of total CD19+ autologous RBD-specific B cells, divided by VOC causing the infection, and COVID-19 disease severity. C) Isotyping of RBD-specific classical, switched MBCs, divided by VOC causing the infection, and COVID-19 severity. D) Spearman correlation between autologous serum neutralization titers and percentage of autologous RBD-specific IgG+ (left panel) and IgM+ B cells (right panel). E) Spearman correlation between days post symptom onset (DPSO) and % RBD-specific IgG (left panel), % RBD-specific IgM (middle panel) and % RBD-specific total MBCs (right panel). (TIF) [file ppat.1012453.s007.tif]

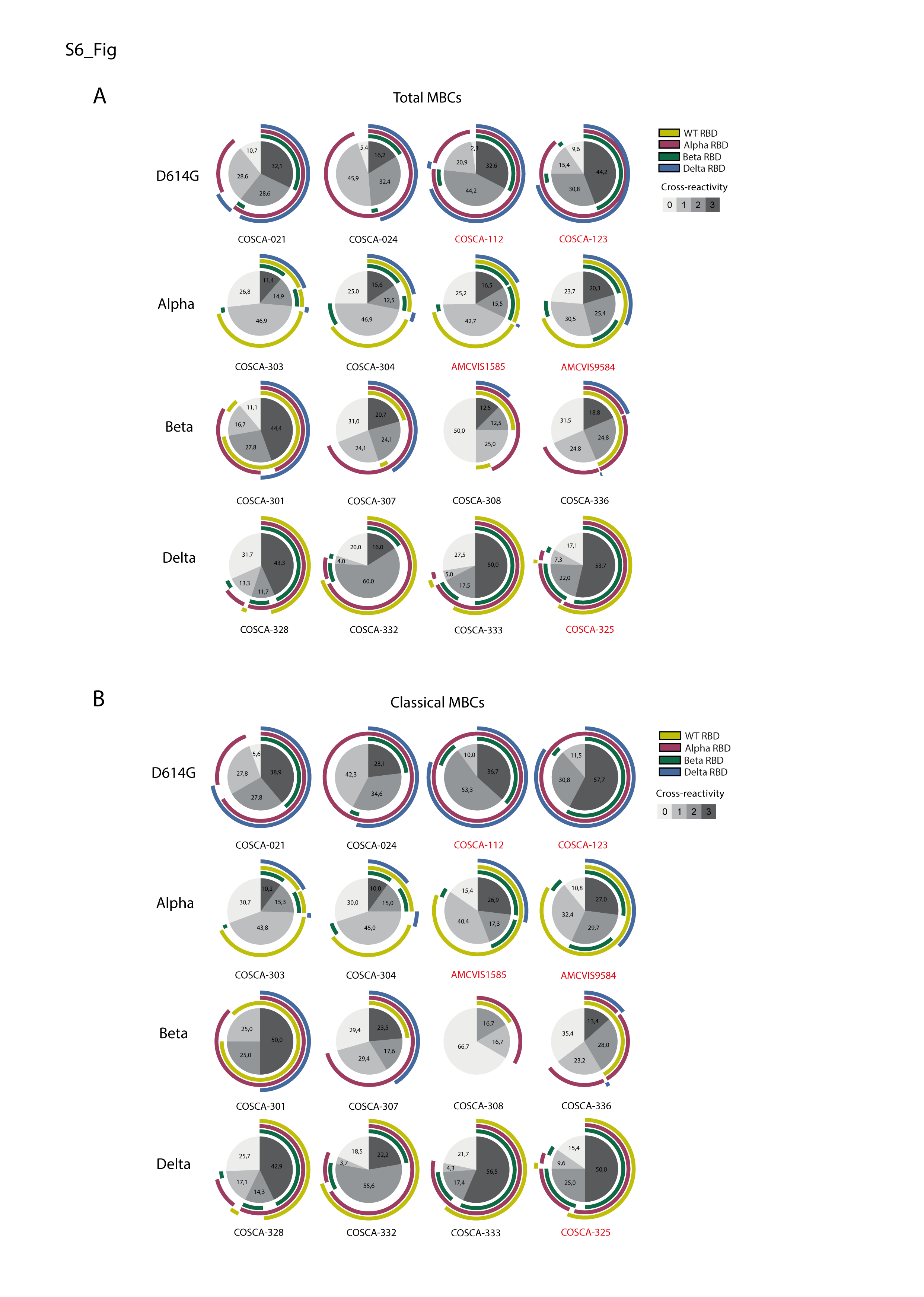

Supplement: S6 Fig — The cross-reactivity legend indicates total MBCs (A) or classical MBCs (B) that recognize the autologous RBD only (0), or bind one, two or three other heterologous RBDs. Patients highlighted in red have been hospitalized. (TIF) [file ppat.1012453.s008.tif]

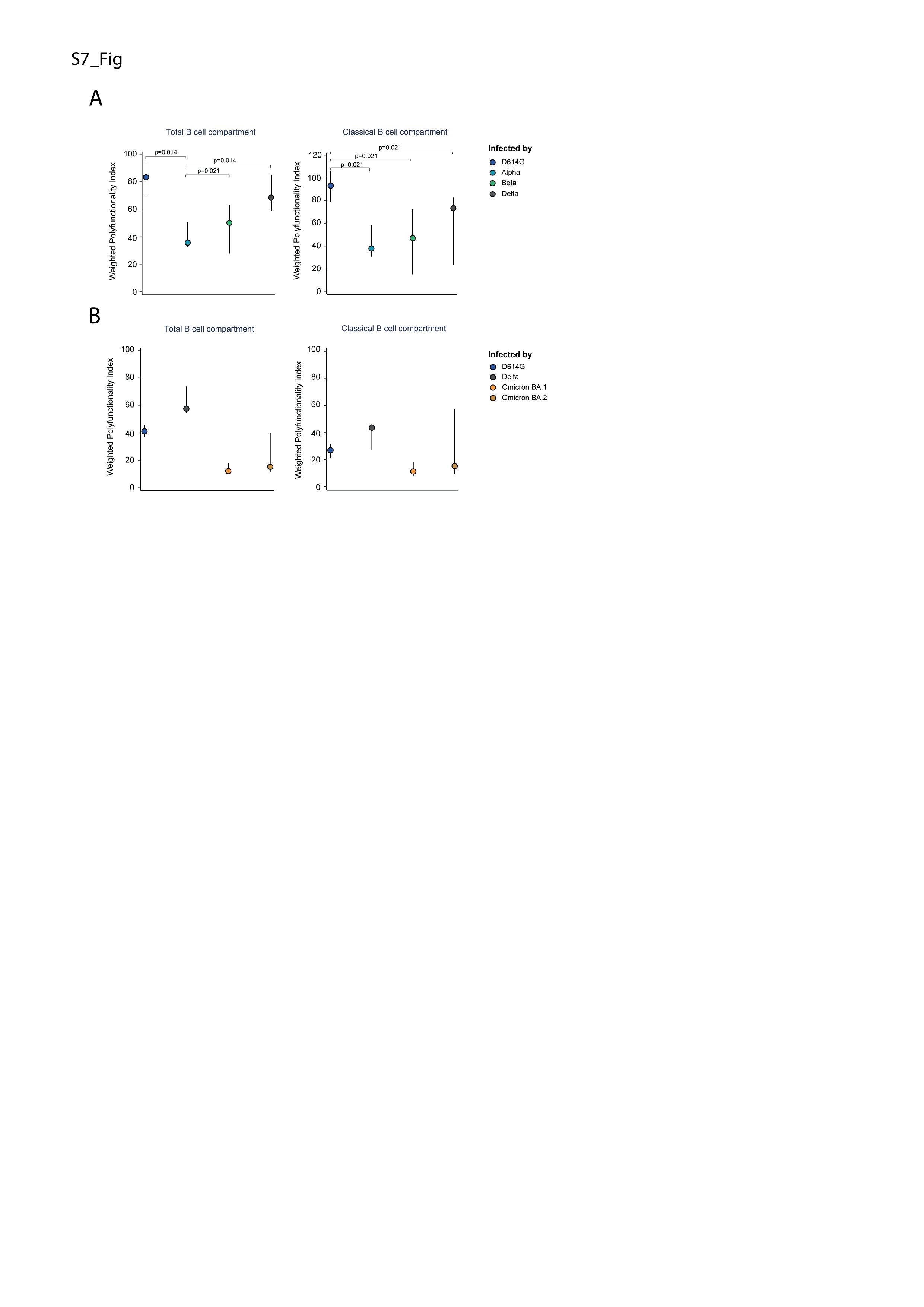

Supplement: S7 Fig — Weighted polyfunctionality indices for the early-pandemic (A) or late-pandemic dataset (B) for the RBD-specific total B cell compartment (left) and classical MBC compartment (right). The indices are calculated taking into account the antigenic distances between the VOCs [18], and compared using a Mann-Whitney U test. (TIF) [file ppat.1012453.s009.tif]

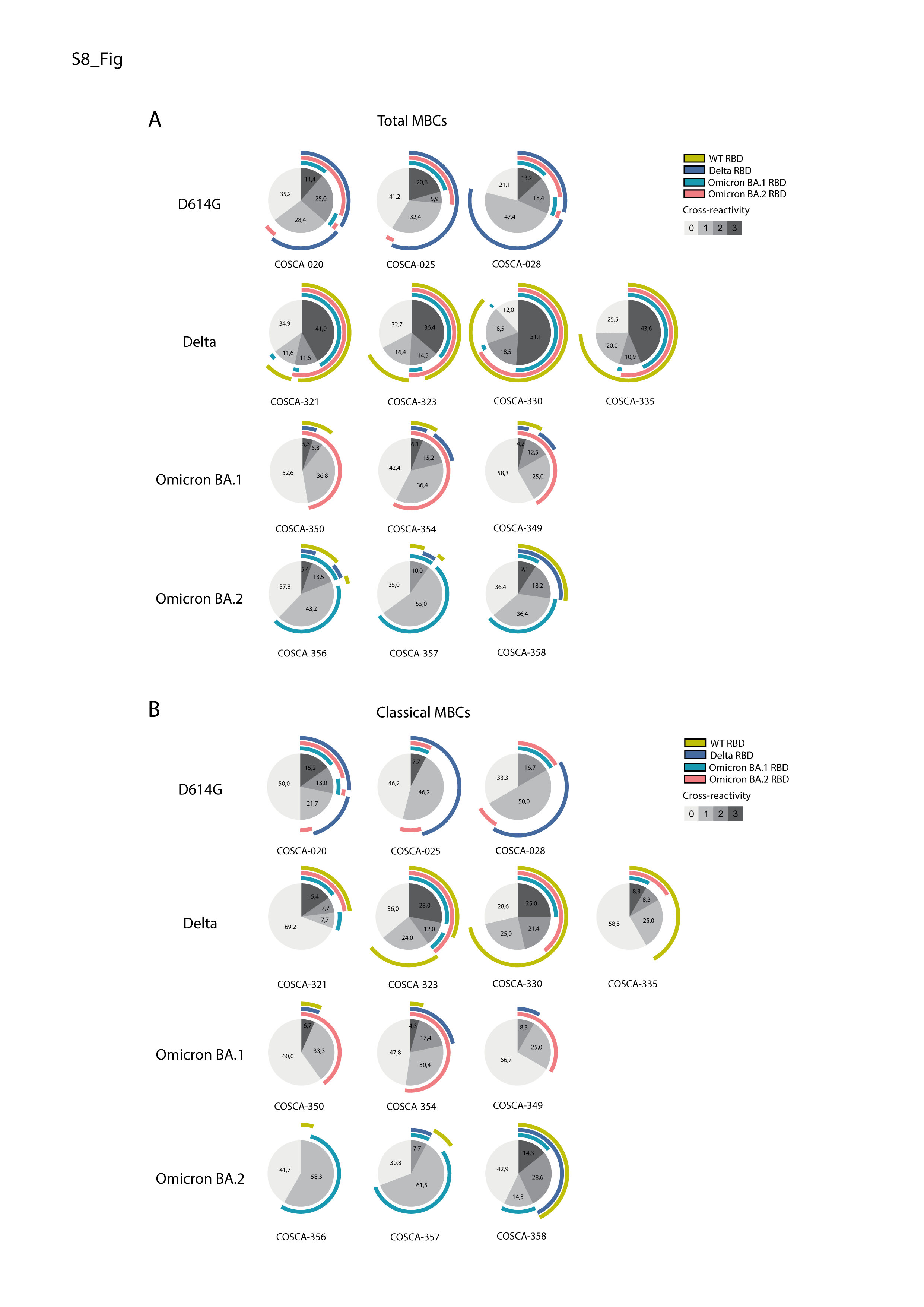

Supplement: S8 Fig — The cross-reactivity legend indicates total MBCs (A) or classical MBCs (B) that recognize the autologous RBD only (0), or bind one, two or three other heterologous RBDs. None of the patients included in this analysis were hospitalized. (TIF) [file ppat.1012453.s010.tif]
